# Modulating the photophysical properties of perylene-based light-harvesting materials *via* bay-induced distortion and core twisting: a combined computational and experimental study

**DOI:** 10.1039/d6ra02167k

**Published:** 2026-07-02

**Authors:** Yousef H. Arafa, Morad M. El-Hendawy, Ahmed Abdelmoneim, Mohamed E. El-Khouly

**Affiliations:** a Nanoscience Program, Faculty of Basic and Applied Science, Egypt-Japan University of Science and Technology New Borg El-Arab City Alexandria 21934 Egypt mohamed.elkhouly@ejust.edu.eg; b Department of Chemistry, Faculty of Science, Sohag University Sohag 82524 Egypt; c School of Chemistry, University of the Witwatersrand 2050 Johannesburg South Africa morad.el-hendawy@wits.ac.za; d Department of Chemistry, Faculty of Science, New Valley University Kharga Egypt

## Abstract

The design of light-harvesting materials is fundamentally rooted in controlling the photophysical and photochemical properties. Herein, we investigate bay-functionalized light harvesting perylenediimides (PDIs) by applying this principle to atomistically defined perylene-based nanographene molecular models. Our approach combines computational modeling (CAM-B3LYP-D3) with a suite of experimental techniques, including steady-state UV-vis absorption, steady-state and time-resolved emission, and electrochemical analysis. The synergistic combination of bay-induced twisting and electron donation is predicted to preferentially destabilize the Highest Occupied Molecular Orbital (HOMO) relative to the Lowest Unoccupied Molecular Orbital (LUMO), activating partial Charge Transfer (CT) character in the primary electronic transition (predominantly HOMO → LUMO). This is visually confirmed by Electron Density Difference (EDD) maps and supported by a calculated increase in dipole moment magnitude (Δ*µ*) vertically and more so adiabatically. This electronic modulation results in a bathochromic shift which can exceed 150 nm in electron-rich derivatives such as PDI-(Py)_2_. In contrast, electron-poor PDI-(CN)_2_ exhibits little to no shift, while the planar, benzimidazole-fused PDI-Imd shows relative HOMO stabilization, leading to a wider HOMO–LUMO gap and displays a notable experimental hypsochromic shift. Ultimately, this work aims to clarify the structure–property relationships of these compounds. By computationally validating how specific structural distortions (twisting) and electronic substituents dictate absorption and emission, we nominate these tailored scaffolds for light-harvesting applications. These insights help uncover the mechanistic rules governing their photophysics, serving as a practical springboard for future studies in perylene-based nanographenes.

## Abstract 2

Tuning the photophysics of perylene diimide derivatives: bay-induced steric distortion and donor coupling activate ICT states (visualized by EDD maps), successfully driving absorption from classic vibronic profiles to deep red-shifted regimes.

## Graphical abstract

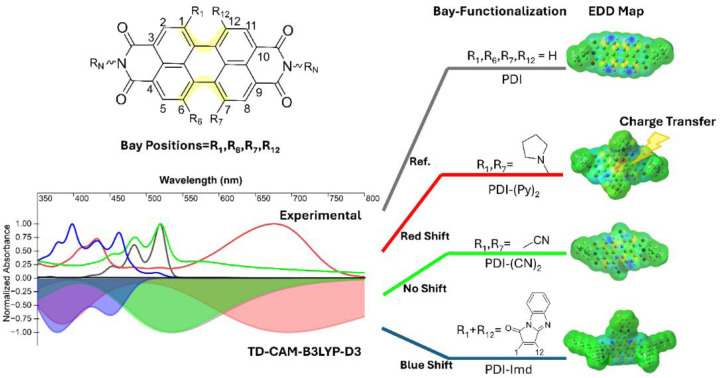

## 1 Introduction

The bottom-up design of functional materials is a cornerstone of modern science, driving innovations in light-harvesting, sensing, and organic electronics.^1^ Perylenediimides (PDIs) have emerged as exceptional building blocks owing to their thermal and photochemical stability, high fluorescence quantum yields, and versatile synthetic modularity.^2,3^ These properties position PDIs as critical components in field-effect transistors and artificial photosynthetic systems.^4,5^ From a modern materials perspective, PDIs serve as molecularly precise, π–π-conjugated chromophores and atomistic substructure models for perylene-based nanographene architectures.^6,7^ Recent studies have highlighted their utility in constructing complex helical and planar nanographenes with tunable electronic properties.^8^

However, the performance of these materials depends heavily on their intrinsic molecular photophysics.^9^ The extended aromatic core of PDIs drives a strong propensity for π–π stacking. While controlled stacking supports ordered supramolecular organization and efficient charge/exciton transport, excessive aggregation induces fluorescence quenching. Consequently, developing strategies to rationally control PDI photophysics remains a key objective in the field.

A highly effective strategy to modulate the core electronic structure of PDIs is bay-position functionalization (positions 1, 6, 7, and 12), as opposed to imide-position modification, which primarily tunes solubility. Introducing substituents at the sterically active bay positions alters the planar PDI core into helically twisted configurations.^10^ This structural distortion reduces molecular symmetry, perturbs frontier orbital distributions, and activates charge-transfer mechanisms. When coupled with electronically active donor or acceptor substituents, bay functionalization promotes partial charge-transfer character and significant spectral shifts,^11–13^ which can modulate emission colors from green to red and suppress aggregation-induced quenching.^14,15^

Previous studies demonstrate that PDI photophysics depends strongly on whether the bay groups act as electron-donating, electron-withdrawing, sterically hindering, or fused/annulated rigidifying moieties.^16–20^ For example, electron-rich, nitrogen-containing bay substituents induce variable HOMO stabilization and intramolecular charge transfer. This results in a bathochromic (red) shift of approximately 20–30 nm compared to bay-unsubstituted precursors.^21^ Conversely, cyano-functionalized PDIs primarily enhance electron affinity and stabilize reduced states, yielding low LUMO levels (−4.3/−4.5 eV).^18^ Interestingly, 1,7-PDI(CN)_2_ derivatives retain the vibronic solution absorption profiles (typically 527, 490, and 459 nm) of the parent PDI, indicating that cyano substitution dictates electron affinity more directly than it induces bathochromic shifts.^20^ Where steric bulk is concerned, bay-alkoxy substitution causes out-of-plane distortion that broadens and red-shifts absorption bands while reducing vibronic resolution. For instance, bulkily substituted 1,12-dialkoxy PDIs exhibit lowest-energy absorption maxima clustered around 582–585 nm red-shifted relative to the parent PDI regardless of chain length; however, their most pronounced systematic effect is water-dependent H-type aggregation, which leads to near-complete fluorescence quenching.^19^

Recent literature underscores the potential of bay-functionalization in tailoring PDIs for advanced technologies. Polar bay-functionalized PDIs support anti-cooperative assembly in water for bioimaging.^22^ In photodynamic therapy, they serve as heavy-atom-free photosensitizers that enable spin–orbit charge-transfer intersystem crossing (SOCT-ISC).^23–26^ In photovoltaics, optimized charge separation and transport in bay-functionalized PDI derivatives have yielded organic solar cells with power conversion efficiencies of 18%.^27^ Beyond these established fields, the tunable energetics of twisted PDIs are driving frontiers in singlet fission^28,29^ and chiral spintronics for broadband polarized light detection.^30^ Building on our laboratory's previous investigations into the aggregation behavior and charge-transfer mechanisms of perylene derivatives,^31–34^ we recognized that a unified framework linking bay-induced twisting to divergent photophysical outcomes—such as the bathochromic shifts in electron-rich twisted systems *versus* the hypsochromic shifts in rigid, planar fused analogues—remains an active area of study.

To address this gap, this work investigates a series designed to isolate these distinct substituent regimes: PDI-(Py)_2_: an electron-rich, donor-twisted bay derivative; PDI-(CN)_2_: an electron-poor, cyano-substituted derivative; PDI-Imd: a rigid, planar benzimidazole-fused analogue. By combining computational Density Functional Theory (DFT) and Time-Dependent DFT (TD-DFT) methods with experimental spectroscopy, we establish a clear structure–activity relationship. We validate a predictive computational model by correlating molecular geometries, frontier orbitals, and dipole magnitude changes with experimental optoelectronic properties and excited-state dynamics. Systematic correlation of these geometric and electronic configurations provides a blueprint for the predictive design of functionalized PDIs, paving the way for advanced light-harvesting systems and molecular nanographene analogs.^35–38^

## 2 Methods and materials

### 2.1 Materials

Investigated light-harvesting systems based on perylene-3,4,9,10-tetracarboxylic diimide namely the PDI parent compound along with the bay-functionalized derivatives: 1,7-dipyrrolidinyl PDI (PDI-(Py)_2_),^38^ 1,7-dicyano PDI (PDI-(CN)_2_),^39^ and the benzimidazole-fused derivative PDI-Imd,^34^ which were obtained from lab stocks, having been prepared according to the standard reported procedures in the cited references. All PDIs were substituted at both *N*-imide positions with cyclopentyl groups, while PDI-Imd featured distinct tridecan-7-yl groups. The purity was verified by thin-layer chromatography (TLC) confirming single spot-homogeneity. Acetonitrile (ACN) and tetrahydrofuran (THF) were purchased at a spectroscopically pure grade from Sigma-Aldrich.

### 2.2 Instruments

UV-vis absorption spectral measurements were carried out on a Shimadzu UV-2600 spectrophotometer at room temperature and with corrected solvent baseline, using fresh solutions by dissolving each PDI derivative in the respective solvent to a dilute concentration on the order of 10^−6^ M that maintains Beer–Lambert law limit maintaining the absorbance below 1 at the wavelength of interest to eliminate aggregation or inner-filter effects. Steady-state fluorescence spectra were recorded on a Shimadzu RF-6000 spectrofluorometer. Fluorescence lifetimes were determined using Time-Correlated Single Photon Counting (TCSPC) model PicoQuant FluoTime 300 spectrometer with a UNISOKUCo., Ltd laser system. Decay curves were analyzed using tail-fitting algorithms by FluoFit software to determine the excited-state lifetimes (*τ*). Cyclic voltammetry (CV) was performed using a Gamry, model ZRA-05087 in oxygen-free THF solvent containing 0.1 M tetrabutylammonium hexafluorophosphate (TBAPF_6_) as the supporting electrolyte. A three-electrode system was employed, consisting of a glassy carbon working electrode, a platinum wire counter electrode, and a Ag/AgCl reference electrode, previously calibrated against the ferrocene/ferrocenium (Fc/Fc^+^) redox couple.

### 2.3 Computational methodology

**Fig. 1:**
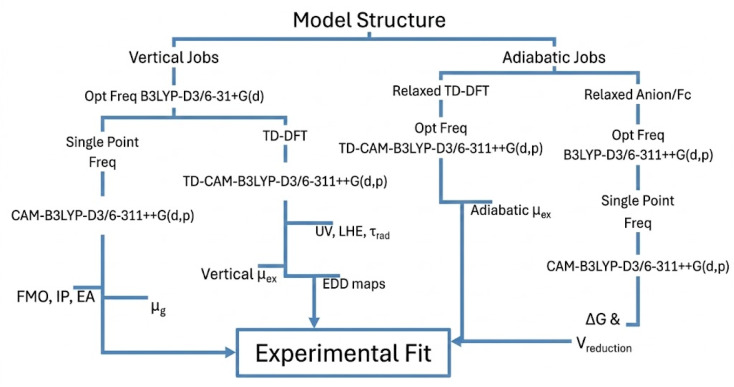
Computational operation flowchart.

Theoretical calculations used DFT and TD-DFT^40,41^ using Gaussian 16, Revision C.01.^42^ GaussView 6 was used for viewing the input and output,^43^ while Chemcraft was employed for molecule drawing with custom aromatic bond representations.^44^ Our approach followed well-established standards for studying photophysics in complex chromophores (Fig. 1).^45,46^ To match our experimental conditions, we selected the implicit Solvation Model based on Density (SMD)^47^ with ACN (polarity: *ε* = 35.7) or THF (polarity: *ε* = 7.58).

#### 2.3.1 Geometry optimization

Initial optoelectronic properties were investigated using the hybrid B3LYP functional incorporating Grimme's D3 dispersion correction (B3LYP-D3)^48,49^ and the 6-31+G(d) basis set.^50^ This level of theory provides a reliable and satisfactory level for optimizing geometries of bulky organic chromophores, including PDI-based light-harvesting systems.^1,51^ After the initial investigation, a relaxed stated TD-DFT study was carried out under the same criteria of the initial vertical TD-DFT study as explained in the next section, where we used CAM-B3LYP-D3/6-311++(d,p) for the S1 geometry optimization under TD-DFT condition. For the later redox study, the radical-anion species of PDI and ferrocene/ferrocenium were optimized in THF at the B3LYP-D3/6-311++G(d,p) level where same-level frequency calculations confirmed the absence of imaginary frequencies. Such optimization provides more accurate diffuse functions to describe anionic species for the calculated thermal free energy correction when converted to match with the experimental value quantitatively in the next step. Finally, we carried frequency calculations on each optimized structure at the same level to confirm we achieved energy minimum on the potential energy surface, proven by zero imaginary frequencies.

#### 2.3.2 Ground and excited states properties

To get sharper electronic properties, we ran single-point energy calculations on the previously optimized geometries for neutral, positive and negative ions at the same ground state geometry to determine the vertical ionization potential (IP) and electron affinity (EA). We decided to select the long-range corrected hybrid functional CAM-B3LYP plus D3 correction,^52^ paired with 6-311++G(d,p). This is justified by CAM-B3LYP design aimed at handling electronic excitations with major charge-transfer (CT) character,^45,53^ where CT states are crucial for PDI systems since bay-functionalization is likely to twist the core-framework and further amplify CT states.^10^ Prior TD-DFT trial of the B3LYP-D3 relegated to the SI (Fig. S1) owing to its inherent self-interaction error (SIE), which leads to systematic underestimation of excitation energies for states with significant CT character. Consequently, we calculated theoretical UV-vis spectra using TD-DFT for the first ten singlet excited states, providing the vertical excitation energies (*E*_ex_) and oscillator strengths (*f*). To additionally analyze the electronic structure and redox behavior, vertical IP and EA values were calculated from CAM-B3LYP-D3/6-31++G(d,p) single-point energies of the neutral, cationic, and anionic species at the same optimized neutral geometry, and hence these values do not include charged-state relaxation. Next, for the redox study, and to estimate the Gibbs free-energy, CAM-B3LYP-D3/6-311++G(d,p) single-point/frequency jobs were then used for the higher-level energetic treatment used to estimate the Gibbs free-energy difference relative to the ferrocene/ferrocenium couple theoretically.^54^ By using GaussView 6 to view the Gaussian 16 output summary the dipole moments were extracted directly, where *µ*_g_ was taken from the optimized ground-state calculation output, *µ*_ex(vertical)_ from the vertical TD-DFT excited-state calculation at the same ground-state geometry, and *µ*_ex(adiabatic)_ from the optimized S1 TD-DFT job outputs. Finally, EDD maps were constructed from the vertical S_0_ → S_1_ TD-DFT calculation by subtracting the ground-state SCF density cube generated using density = scf from the excited-state density cube generated using density = CI, then mapping the resulting difference-density cube on the SCF density surface in GaussView 6 using the stated isovalue.

## 3 Results and discussion

### 3.1 Geometric and electronic properties

**Fig. 2:**
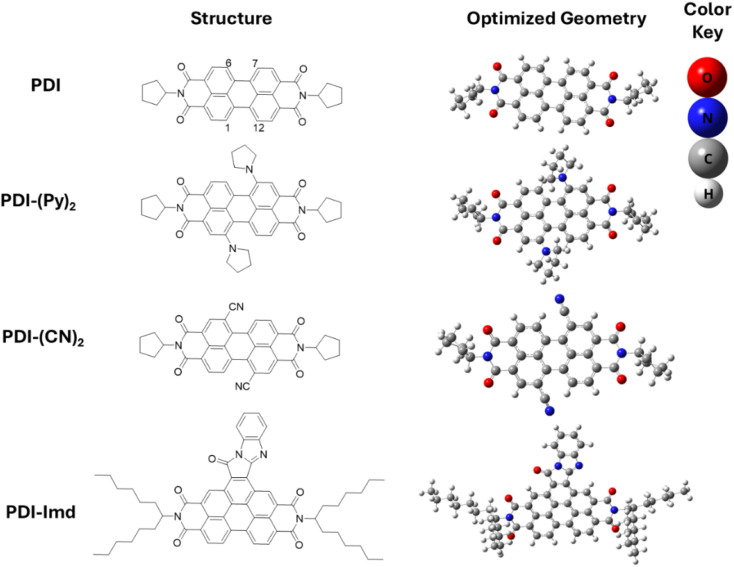
Structural diagram of investigated parent and bay-functionalized PDI-based light-harvesting systems and their DFT optimized geometries using B3LYP-D3/6-31+G(d).

**Fig. 3:**
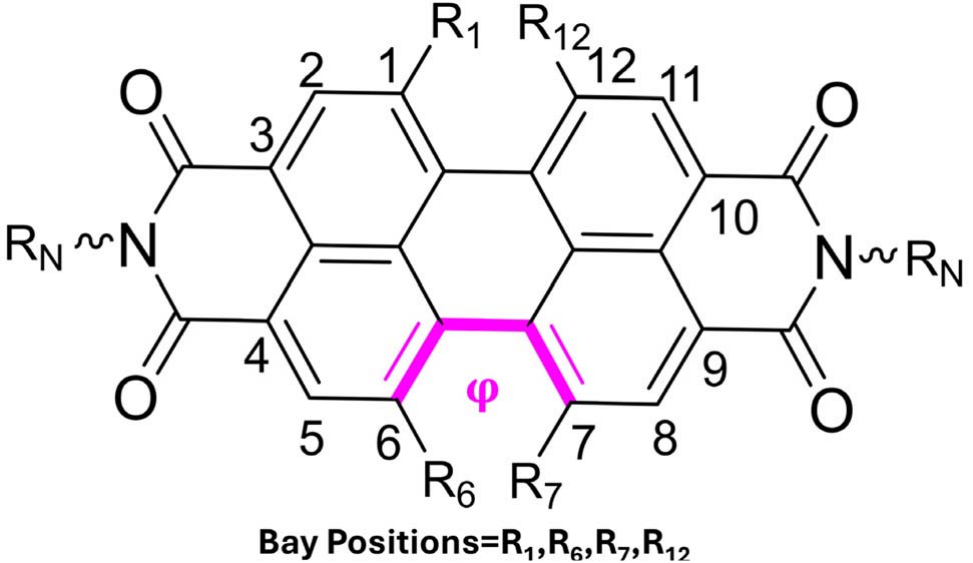
General structural depiction of the dihedral twist angle *φ* within the polycyclic framework.

To elucidate the structure–property relationships governing the PDI scaffold, we first examined the optimized ground-state geometries (Fig. 2). A key geometric parameter influenced by this substitution is the dihedral angle (*φ*) of the PDI framework (Fig. 3). These structural parameters are listed in Table 1, which includes the dipole moments (*µ*) for the ground, vertical, and adiabatic excited states to highlight the connection between geometry and electronic polarization.

**Table 1:** Dihedral angle [*φ*, (°)] between the naphthalene moieties of the investigated molecules in the ground state coinciding with the dipole moment magnitude (Debye) change between the ground, vertical and relaxed excited state

Structure	PDI-(Py)_2_	PDI-(CN)_2_	PDI-Imd	PDI
Parameter				
*φ*	21 °	18° a	0°	0 °
*µ*_g_	6.91	3.15	2.93	0.07
*µ*_ex(vertical)_	8.58	3.13	3.03	0.08
Δ*µ*_(vertical)_	1.67	−0.02	0.10	0.01
*µ*_ex(adiabatic)_	9.35	3.13	4.15	0.23
Δ*µ*_(adiabatic)_	2.44	−0.02	1.22	0.16

a Note: DFT optimization overestimates the dihedral twist for PDI-(CN)_2_ as an isolated molecule energy minimum at 18° within the SMD continuum. As established by high-level *ab initio* and DFT benchmarks in the literature, a twist of 16°–19° can be observed in comparison to single-crystal X-ray diffraction (XRD) and condensed-phase experimental data^58^ support that the linear, low-steric profile of cyano groups allows the core to remain nearly planar on average in dynamic solution or condensed phases. See Discussion for structural/electronic implications.

The parent PDI adopts an expectedly planar *D*_2h_ geometry, consistent with literature precedents.^55,56^ Notably, introducing functional substituents at the bay positions induces a twisting of the central PDI framework. This distortion is quantified by the core dihedral angle (*φ*) between the two naphthalene moieties. As summarized in Table 1, the optimized geometries exhibit near-identical dihedral angles on both sides of the core, which have been rounded to the nearest integer. This is consistent across the investigated systems with the bulky substituents responsible for evident steric hindrance.^10,57^ PDI-(Py)_2_ showed maximum core twisting *φ* = 21°, then PDI-(CN)_2_ at 18°, whereas the benzimidazole-fused PDI is planar. The steric bulk of the substituents forces the naphthalene units out of plane, resulting in a twisted polycyclic core of PDI (Fig. 3), While DFT calculations predict an 18° core twist for PDI-(CN)_2_ in the SMD environment, established crystallographic and theoretical benchmarks^58^ confirm a nearly planar structure (*φ* = 5°) in condensed phases owing to the linear geometry and low-steric profile of the –CN groups, which allow environmental forces to easily overcome the shallow torsional barrier. Therefore, the PDI-(CN)_2_ and benzimidazole-fused PDI effectively act as planar scaffolds experimentally, sharply contrasting with the bulky-group-distorted PDI-(Py)_2_. The strict co-planarity of the fused PDI-Imd provides a significant geometric baseline to isolate the electronic effects of fusing a heteroaromatic ring directly to the PDI-core, as discussed later.

**Fig. 4:**
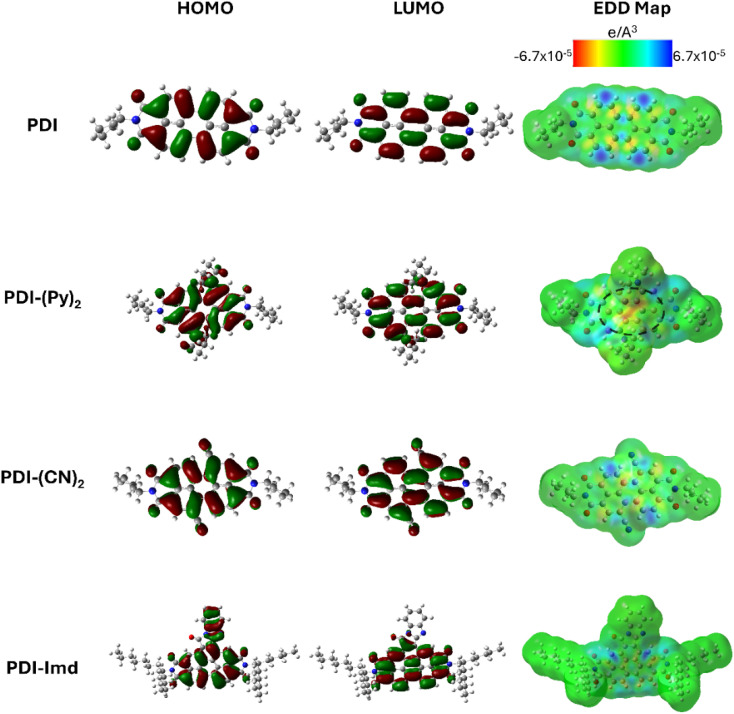
FMO and EDD map of the studied light harvesting molecular systems.

Steric considerations induce significant changes in the frontier molecular orbitals (FMOs), specifically the Highest Occupied and Lowest Unoccupied Molecular Orbitals (HOMO and LUMO) as shown in Fig. 4, which in turn affect the optoelectronic behavior. For the derivative with electron-donating pyrrolidinyl groups PDI-(Py)_2_, FMOs show the substituents contribute atomic orbital density to the HOMO level distribution; this is in line with the support of nitrogen atoms for extended conjugation from the central PDI framework. Nitrogen-based donors have been found to excel at extending the core's electronic conjugation.^11,12^

In support of this assessment, electron density difference maps (EDD Maps) as shown in Fig. 4 visualize this transfer with electron density (e Å^−3^) flowing during excitation. All EDD Maps were plotted with an isosurface value set to ± 10^−4^ and indicate a decrease in electron density localized on the donor region (yellow area) *versus* the electron density increasing in acceptor region (blue area). Interestingly for PDI-(Py)_2_, a strong contrast is shown for the pyrrolidinyl-containing region (green/blue) compared to the PDI-core (yellow/red) which was regionally highlighted in Fig. 4.

**Fig. 5:**
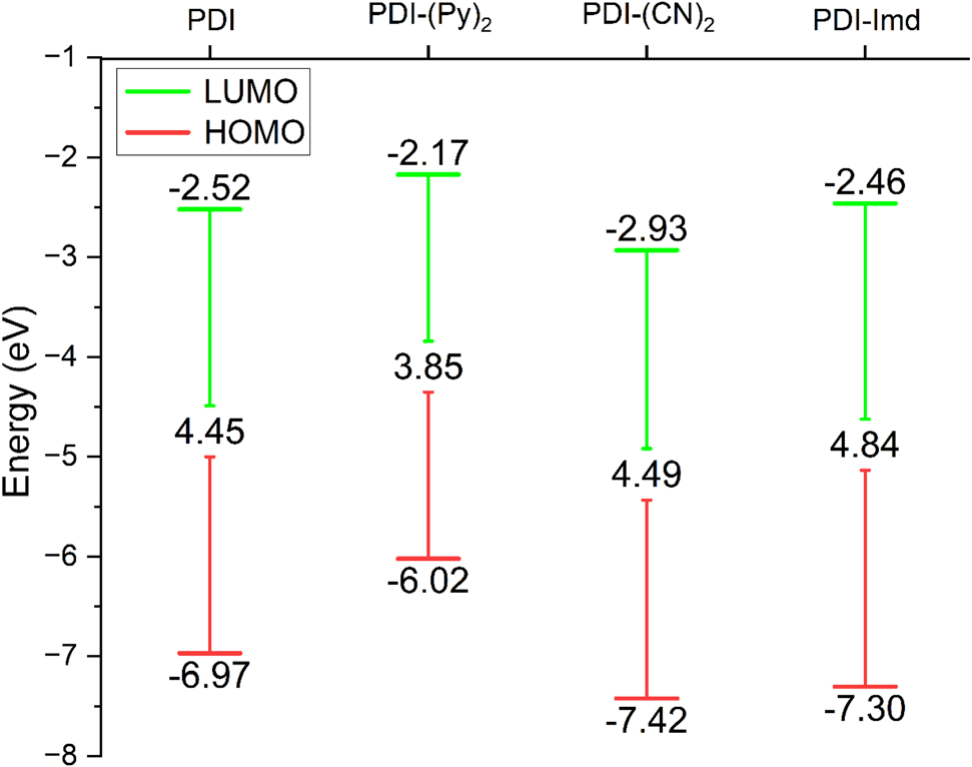
Energy level diagram of the calculated HOMO (red) and LUMO (green) energies (in eV) and the resulting *E*_gap_ for the PDI derivatives.

This steric, donor-induced twisting preferentially destabilizes the HOMO, making the 1,7-positions highly sensitive to electronic substitution and well-suited for donor–acceptor exchange. This is evidenced by a substantial +0.95 eV destabilization of the HOMO energy to −6.02 eV for PDI-(Py)_2_, relative to the planar parent PDI's −6.97 eV, narrowing the HOMO–LUMO gap to 3.85 eV, as shown visually in Fig. 5. This preferential HOMO destabilization, driven by the broken orbital degeneracy in the twisted core, aligns with literature reports on related bay-functionalized rylenes and theoretically accounts for the observed red-shift in the UV-vis absorption spectrum.^10,58^ Conversely, the planar PDI-Imd exhibits a widened HOMO–LUMO gap of 4.84 eV compared to the parent PDI (4.45 eV). This widening is driven primarily by the stabilization of the HOMO level, providing a clear theoretical rationale for the observed hypsochromic (blue) shift.

### 3.2 Experimental validation

#### 3.2.1 UV-vis absorption & electrochemistry

**Fig. 6:**
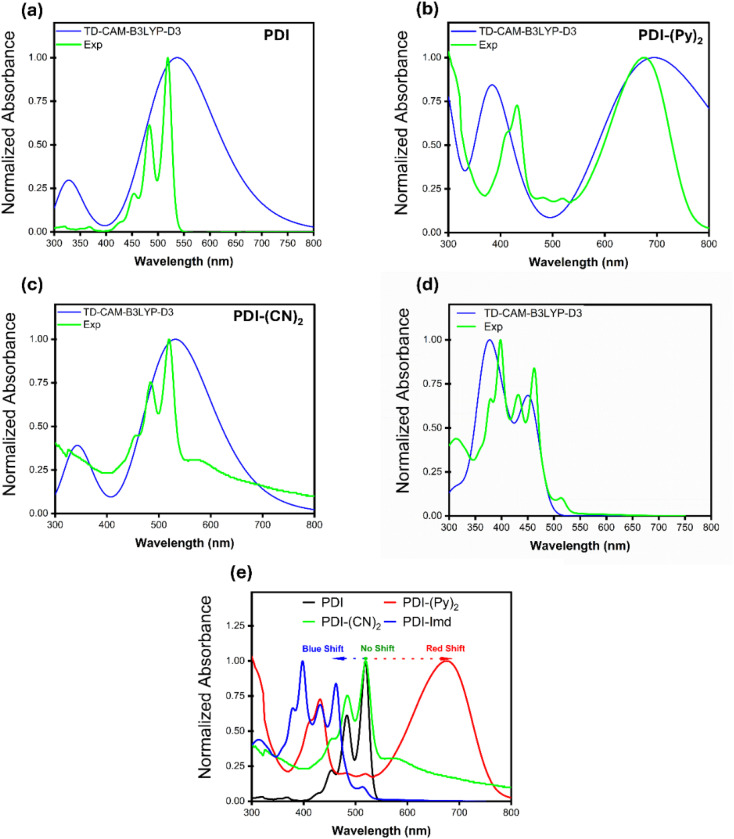
Overlay of normalized experimental and TD-CAM-B3LYP-D3 calculated absorption spectra of each of the investigated derivatives (a–d) and experimental spectral shifts upon bay-functionalization (e) in ACN.

Evident from the FMO energy gap, tuning it should directly affect absorption characteristics. The experimental UV-vis spectra in ACN, as in Fig. 6, presents a unique profile variation, with the key photophysical parameters presented in Table 2 using TD-DFT output and eqn (1) to calculate Light Harvesting Efficiency (LHE). The planar parent PDI's, as well as the effectively planar PDI-(CN)_2_ and PDI-Imd spectra have a well-resolved vibronic fine structure, showing the 0–0 and 0–1 transitions typical of rigid, planar chromophores in accordance with the Franck–Condon principle owing to vibrational coupling showing experimental corroboration that PDI-(CN)_2_ maintains a planar core in solution. Indeed, such structured spectral profiles are a hallmark of large, rigid sp^2^-hybridized aromatic systems, where the electronic transitions are directly coupled to vibrational modes, a phenomenon we have previously analyzed in substituted anthracenes.^59^

**Table 2:** The spectral properties of examined PDI molecular structures, computed at the TD-CAM-B3LYP-D3/6-311++G(d,p) level^b^

Structure	State	*E*_ex_ (eV)	*λ* ^calc^_max_ (nm)	*λ* ^exp^_max_ (nm)	*f*	LHE	ΣLHE	Major contributing configuration^a^	Contribution (%)
PDI	S_o_ → S_1_	2.31	537	518	1.27	0.95	1.53	HOMO → LUMO	98
	S_o_ → S_3_	3.78	328		0.38	0.58		HOMO_−2_ → LUMO	83
PDI-(Py)_2_	S_o_ → S_1_	1.78	695	677	0.90	0.88	2.46	HOMO → LUMO	98
	S_o_ → S_4_	3.24	383		0.70	0.80		HOMO_−2_ → LUMO	88
	S_o_ → S_9_	4.25	291		0.65	0.78		HOMO → LUMO_+8_	34
PDI-(CN)_2_	S_o_ → S_1_	2.33	532	520	1.08	0.92	1.52	HOMO → LUMO	98
	S_o_ → S_3_	3.61	344		0.40	0.60		HOMO_−2_ → LUMO	80
PDI-Imd	S_o_ → S_1_	2.74	453	462	1.02	0.91	3.06	HOMO → LUMO	90
	S_o_ → S_3_	3.09	401		0.74	0.82		HOMO_−1_ → LUMO_+1_	44
	S_o_ → S_4_	3.35	370		1.27	0.95		HOMO → LUMO_+1_	46
	S_o_ → S_6_	3.96	313		0.21	0.38		HOMO_−2_ → LUMO_+1_	85

a The listed configurations represent the major individual contributions from the TD-DFT output. Therefore, high-energy states with modest contribution values, such as the S0 → S9 state of PDI-(Py)_2_, should be considered mixed states rather than pure one-electron transitions.

b Abbreviations: *E*_ex_ = vertical excitation energy; *λ*^calc^_max_ = calculated absorption maximum; *λ*^exp^_max_ = experimental absorption maximum; *f* = oscillator strength; LHE = light-harvesting efficiency; ΣLHE = sum of LHE values for the listed oscillator-strength-bearing transitions; contribution (%) = contribution of the listed major electronic configuration.

For PDI-(Py)_2_, the calculated *λ*_max_ (695 nm) is in excellent agreement with the experimental value of 677 nm. Additionally, the model correctly captures the general changes in spectral shape as it overlaps the well-resolved vibronic structure for the planar PDI and PDI-Imd systems, and it accurately reproduces the broad, featureless absorption band for the twisted PDI-(Py)_2_. Furthermore, the model successfully predicts the blue-shift for PDI-Imd, calculating a *λ*_max_ of 453 nm which is in good agreement with the experimental *λ*_max_ of 462 nm, which is experimentally shifted by a moderate 56 nm shift relative to the parent PDI as shown in Table 2.

The theoretical model was successfully validated by comparing the experimental absorption spectra with the TD-DFT UV-vis results (Fig. 6 and Table 2), which demonstrated minimal error (<20 nm). Notably, the long-range-corrected CAM-B3LYP-D3 functional yielded *λ*_max_ values and overall spectral profiles that successfully captured the presence or absence of vibronic features across the entire series. This stands in sharp contrast to standard B3LYP-D3 (Fig. S1), which failed to replicate these trends satisfactorily.

A key point of validation is the successful reproduction of the observed bathochromic shift for PDI-(Py)_2_, which lines up with previous reports of large red-shifts in PDIs which were bay-functionalized with electron-donating groups.^11,60^ Compared to the parent PDI, a large bathochromic shift is observed for PDI-(Py)_2_ corresponding to an increase by ∼158 nm compared to the parent PDI *λ*^Exp^_max_ value listed in Table 2.

The LHE of this primary transition is high ranging from 0.88 to 0.92 shown in Table 2. Notable systems with multiple higher-energy transitions with significant oscillator strengths include PDI-(Py)_2_ and PDI-Imd where the largest sums of LHE (ΣLHE) are found to be 2.46 and 3.06, respectively.^61^

These results indicate that the investigated bay functionalized molecular models reveal strong oscillator-strength-bearing transitions, but it must be noted that they enhance different aspects of light harvesting. Pyrrolidinyl functionalization has the clearest effect in red/NIR-shifted light harvesting due to combining the electronic coupling of the donating-nitrogen with bay-induced core twisting, hence it leads our studied molecular models in shifting the experimental absorption maximum of PDI-(Py)_2_ to 677 nm. By contrast, PDI-(CN)_2_ and the parent PDI retain the same absorption character with nearly similar light-harvesting capacity. Interestingly, PDI-Imd shows a high cumulative ΣLHE at the blue-shifted principal absorption due to its planar fused structure, consistent with short-axis annulated PDI systems where fusion can increase the optical/HOMO–LUMO gap and induce hypsochromically shifted absorption.^17,21^

This interpretation is also consistent with a qualitative prediction for bulky nonconjugated bay substituents such as *tert*-butyl groups. However, from a theoretical molecular electronic perspective, steric bulk alone should not be expected to show the remarkable HOMO destabilization, excited-state dipole increase, or red/NIR bathochromic shift observed for PDI-(Py)_2_, because *tert*-butyl is not a strongly π-conjugated nitrogen donor. Therefore, *tert*-butyl-like substitution would likely be expected to increase steric congestion around the PDI core which perturbs planarity and on a macro scale is consistent with reducing close π–π stacking to control aggregation, solubility, and morphology *versus* generating the donor-twist charge-transfer response observed for pyrrolidinyl substitution.^16,19,62^

1 LHE = 1 − 10^−*f*^

Calculations reveal the S_0_ → S_1_ transition, predominantly composed of the HOMO → LUMO excitation, alongside several higher-energy excited states with significant oscillator strength (*f*), as shown in Table 2. Higher transitions may contribute to overall absorption cross-section. However, Kasha's rule supports that rapid internal conversion means subsequent photophysical events, such as fluorescence, will follow predominantly from the lowest-lying singlet excited state (S_1_) which predominates with a HOMO–LUMO. Hence, this energy harvested by absorption may dissipate efficiently through radiative decay pathways.^2,63^

**Fig. 7:**
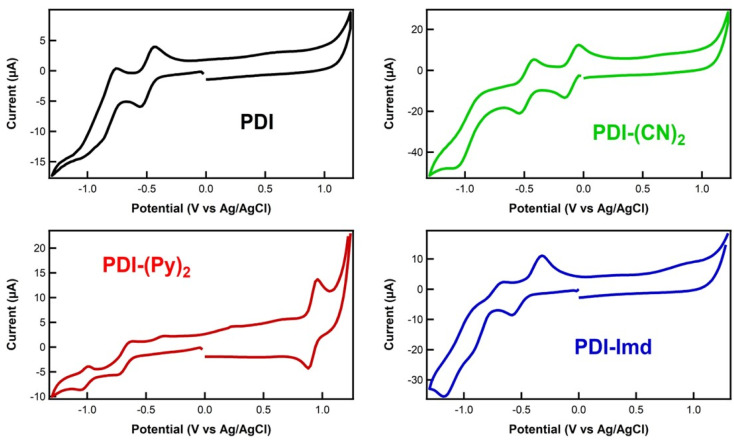
Cyclic voltammogram of examined PDIs (1.0 × 10^−4^ M) measured in THF with Ag/AgCl as reference electrode with scan rate 50 mV s^−1^ at 298 K.

To investigate the individual contributions of the frontier molecular orbitals (FMOs) to the narrowing of the optical gap, we turned to cyclic voltammetry (CV) (Fig. 7). This approach offers a direct experimental probe of the HOMO energy levels across the bay-functionalized series, serving as an empirical counterpoint to the DFT predictions.

By analyzing the reduction potentials, which are negative potentials, the electronic nature for bay-functionalization is revealed. The electron-poor PDI-(CN)_2_ exhibits the least negative reduction onset at −0.09 V, proving a stabilized LUMO level and enhanced electron affinity. Conversely, the electron-rich PDI-(Py)_2_ exhibits the most negative first reduction potential of −0.67 V, confirming the destabilizing influence of the donor groups on the LUMO levels.

In line with previous FMO trends PDI-(Py)_2_ exhibited a distinct oxidation potential at 0.92 V *vs.* Ag/AgCl. The observed low oxidation potential confirms synergistic electronic-steric interplay. While dialkylamino functional groups naturally exhibit reversible oxidation potentials due to intrinsic mesomeric electron donation by nitrogen's lone pair, the corresponding sterically induced core twist in PDI-(Py)_2_ disrupts the extended π-conjugation of the perylene core. This structural distortion amplifies the electronic effect and further destabilizes the HOMO level beyond what electronic donation alone would achieve. On the other hand, the planar, electron-deficient PDI-(CN)_2_, PDI-Imd and the parent PDI showed no discernible oxidation peaks within the accessible solvent (THF) window. Because PDI-(CN)_2_ retains a planar geometry in solution, it avoids the sterically induced HOMO destabilization seen in PDI-(Py)_2_, allowing the overwhelming electron-withdrawing inductive effect of the –cyano groups to keep the HOMO level deeply stabilized. This absence of oxidative current is a typical phenomenon documented for relatively electron deficient PDIs which confirm their HOMO levels lie significantly deeper in energy.^17,64^

To further analyze these electronic trends, we moved beyond qualitative comparisons to a quantitative validation of the CAM-B3LYP model. Hence, the theoretical redox potentials were studied upon being derived from the computational output relative to the (Fc/Fc^+^) couple. As defined in eqn (2) to cancel systematic solvation errors:^54^2
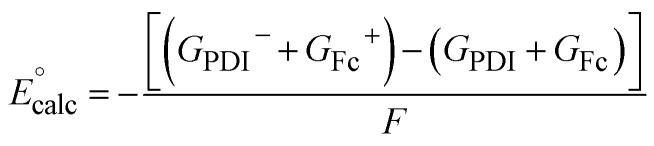
where *G* denotes the calculated Gibbs free energy of the respective neutral or charged species in solution, and *F* is the Faraday constant. Considering the comparison to the experimental potentials, they were standardized to the ferrocene scale (*E*^corr^_Exp_) utilizing eqn (3):

3 *E*^corr^_Exp_ = *E*^raw^_Exp_ − 0.604 V

where *E*^raw^_Exp_ is the measured potential against the Ag/AgCl reference and 0.604 V represents the potential shift relative to ferrocene in THF.^65^ As depicted in Table 3, a good agreement between calculated and experimental reduction potentials with a Mean Absolute Error (MAE) of less than 0.1 V. Only a slight cathodic deviation of 0.13 V is predicted for PDI-(Py)_2_, which is attributed to the irreversible nature of the reduction wave experimentally showing a peak potential (*E*_pc_) shifted anodically by kinetic factors relative to the thermodynamic standard potential (*E*°) results by DFT.^66^ Such accurate alignment supports the accurate description by the FMO energies for the computationally studied planar and twisted systems.

**Table 3:** Quantitative validation of computed reduction potentials against experimental data standardized to the ferrocene scale^a^

Structure	*E*_calc_ (V *vs.* Fc)	*E* ^raw^_Exp_ (V *vs.* Ag/AgCl)	*E* ^corr^_Exp_ (V *vs.* Fc)	MAE (Δ*V*)
PDI	−1.01	−0.44	−1.04	0.03
PDI-(Py)_2_	−1.40	−0.67	−1.27	0.13
PDI-(CN)_2_	−0.65	−0.09	−0.69	0.04
PDI-Imd	−1.01	−0.45	−1.05	0.04

a Abbreviations: *E*_calc_ = calculated reduction potential *versus* Fc/Fc^+^; *E*^raw^_Exp_ = experimental reduction potential measured *versus* Ag/AgCl; *E*^corr^_Exp_ = experimental reduction potential corrected to the Fc/Fc^+^ scale; MAE = mean absolute error between calculated and corrected experimental potentials.

#### 3.2.2 Steady-state and time-resolved emission

**Fig. 8:**
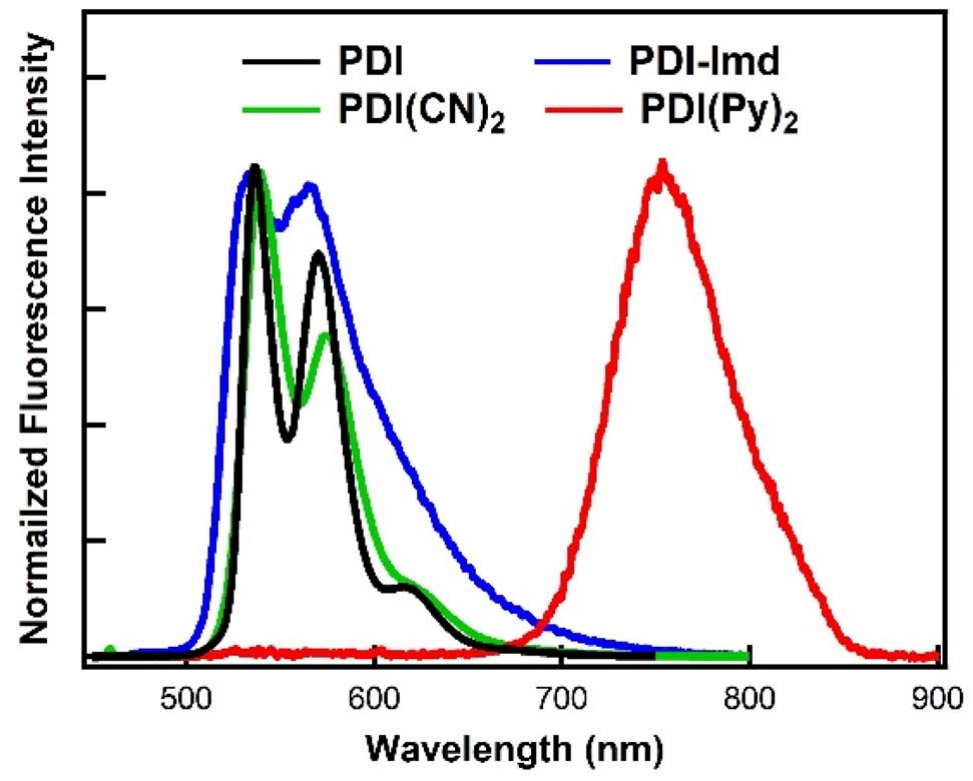
The normalized fluorescence emission spectra of the investigated PDI derivatives in MeCN at room temperature; *λ*_ex_ = 470 nm.

Due to the theoretical prediction of a relatively large change in dipole moment magnitude (Δ*µ*_(vertical)_ = 1.64 Debye; Δ*µ*_(adiabatic)_ = 2.44 Debye) for PDI-(Py)_2_ suggesting electronic reorganization in the excited state. A comparative overview is featured in Fig. 8 as the spectral data could reveal a quantitative contrast between the planar and twisted molecular structures, the former is consistent with the established photophysics of the polycyclic perylene core of PDIs.^2,63^

The planar parent PDI and electron-poor PDI-(CN)_2_ exhibit characteristic mirror-image emission profiles with well-resolved vibronic fine structures, whereas the heteroatom-fused PDI-Imd displays a broader, less-resolved band. In acetonitrile (MeCN), PDI-(CN)_2_ features distinct emission maxima at 537 and 573 nm, corresponding to the 0–0 and 0–1 transitions, respectively, which mirror its steady-state absorption features.

In stark contrast, the bay-functionalized PDI-(Py)_2_ exhibits a fundamentally altered profile; its vibronic structure is entirely lost and replaced by a broad, featureless emission band centered at 772 nm in MeCN. This represents a substantial bathochromic shift of >230 nm relative to the emission of both the parent PDI and PDI-(CN)_2_.

**Fig. 9:**
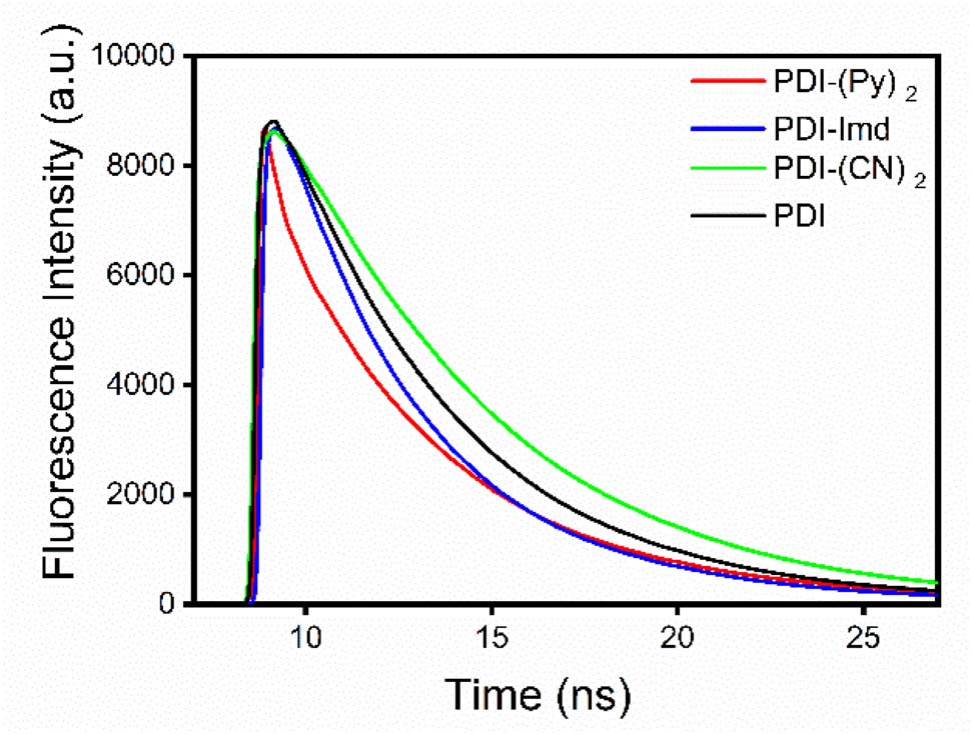
Fluorescence decay profiles of the investigated PDI derivatives in MeCN at room temperature; *λ*_ex_ = 442 nm, measured at the fluorescence maxima of each compound.

To evaluate the excited-state dynamics, time-correlated single-photon counting (TCSPC) measurements were performed (Fig. 9). The fluorescence lifetimes (*τ*_f_) in MeCN were determined to be 3.12 ns for PDI, 3.85 ns for PDI-(CN)_2_, and 2.53 ns for PDI-Imd. Interestingly, PDI-(Py)_2_ exhibited a biexponential decay profile with lifetimes of 0.30 ns and 3.04 ns. This non-monoexponential decay under polar conditions is consistent with enhanced, multi-channel excited-state relaxation pathways active in the donor-substituted, twisted core. Notably, these experimental lifetime values are utilized here to establish a comparative time-resolved trend rather than a rigorous, direct kinetic deconvolution.

To assess the decay mechanism, the theoretical radiative lifetime (*τ*_rad_) was estimated using the simplified Einstein A coefficient using eqn (4):^67^4
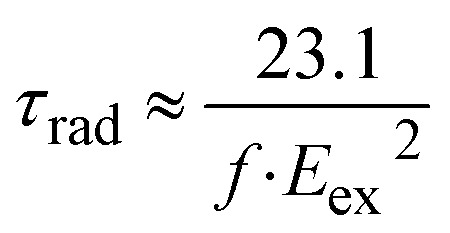
where *f* is the oscillator strength and *E*_ex_ is the vertical excitation energy (eV). As shown in Table 4, it is revealed by comparison that the relatively twisted PDI-(Py)_2_ shows a longer calculated lifetime of *τ*_rad_ ≈ 8.1 ns, while more rigid analogs show better agreement. Since *τ*_exp_ = 1/(*k*_rad_ + *k*_non-rad_) and *τ*_rad_ = 1/*k*_rad_, having such notable discrepancy between the theoretical radiative lifetime and the observed experimental lifetimes supports a significant contribution from non-radiative relaxation of non-radiative relaxation (*k*_non-rad_ ≫ *k*_rad_) where highly efficient internal conversion and ICT pathways thoroughly outcompete radiative decay.

**Table 4:** Comparison of approximate theoretical radiative-lifetime benchmarks (*τ*_rad_) and experimental (*τ*_exp_) experimental fluorescence lifetimes from ACN TCSPC measurement^a^

Structure	*f*	*E*_ex_ (eV)	*τ*_rad_ (calc, ns)	*τ*_exp_ (ns)
PDI	1.27	2.31	3.4	3.12
PDI-(Py)_2_	0.90	1.78	8.1	0.30, 3.04^b^
PDI-(CN)_2_	1.08	2.33	3.9	3.85
PDI-Imd	1.02	2.74	3.0	2.53

a Abbreviations: *τ*_rad_ = approximate calculated radiative lifetime estimated from oscillator strength and excitation energy; *τ*_exp_ = observed fluorescence lifetime obtained from TCSPC decay fitting in ACN.

b For PDI-(Py)_2_, listed values are empirical decay components.

### 3.3 Computational analysis of charge-transfer character

Given the experimental evidence for significant geometric relaxation in the excited state, assessing the resulting partial CT character in the primary S_0_ → S_1_ electronic transition is necessary. In this case, it may be closely tied to the partial Intramolecular Charge Transfer (ICT) model. Structural geometric distortion during excitation, which in turn may equilibrate to a highly polar ICT state breaking electronic symmetry and causing electrons to separate between the donor-substituent and acceptor-core.^10,12,61^

CT characters may be quantified using the dipole moment magnitude change upon photoexcitation (Δ*µ*). Table 1 has shown that twisted PDI-(Py)_2_ has a calculated vertical Δ*µ* of 1.67 Debye which increased upon geometry relaxation in the S_1_ state to 2.44 Debye. This magnitude is consistent with recent electroabsorption measurements for bay-substituted PDIs.^68^ This increase quantitatively postulates electron density redistribution and charge separation in addition to Localized Excitation (LE), surpassing the parent PDI which had a negligible change (Δ*µ* ≈ 0 Debye) indicative of pure LE states. For PDI-(CN)_2_, the small negative Δ*µ* value of −0.02 D is best regarded as a near-zero change within this series, indicating that excitation does not significantly increase molecular polarity relative to the already polarized cyano-substituted ground state. Additionally, for PDI-(Py)_2_ Δ*µ*_adiabatic_ = 2.44 Debye is double that of the asymmetric PDI-Imd's 1.22 Debye, further confirming that core twisting increases polarity significantly beyond the baseline of geometric asymmetry.

Deriving from the electronic energy of neutral, positive and negative ions, the vertical IP and vertical EA respectively were calculated, further we calculated both vertical chemical hardness (*η*) and electronegativity (*χ*) as shown in Table 5; using eqn (5)–(8). These results provide a final, independent layer of quantitative descriptors which translate complex electronic structure into chemically intuitive parameters.

5 IP = *E*_cation_ − *E*_neutral_

6 EA = *E*_neutral_ − *E*_anion_

7
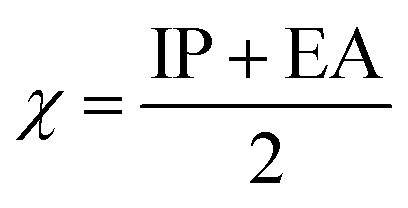
8
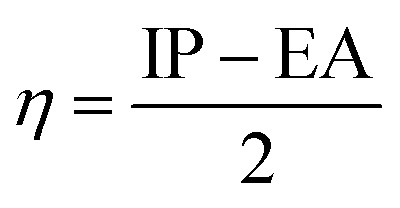


**Table 5:** Quantum chemical descriptors of the investigated PDI molecular systems computed using the CAM-B3LYP-D3/6-311++G(d,p) model chemistry

Parameter	IP (eV)	EA (eV)	*η* (eV)	*χ* (eV)
Structure				
PDI	5.82	3.59	1.11	4.71
PDI-(Py)_2_	4.90	3.22	0.84	4.06
PDI-(CN)_2_	6.29	4.01	1.14	5.15
PDI-Imd	6.22	3.50	1.36	4.86

According to the Hard and Soft Acids and Bases (HSAB) theory, chemical hardness (*η*) measures a molecule's resistance to electron configuration change. In this context, resistance to charge separation.^69^ The calculated hardness for PDI-(Py)_2_ (*η* = 0.84 eV) is the lowest in the series signifying, according to HSAB theory, a minimal energy penalty for separating charge, quantitatively confirming its propensity to form a CT state. Conversely, PDI-Imd shows the highest chemical hardness (*η* = 1.36 eV), indicating the greatest resistance to charge separation and reinforcing its assignment as a LE state.

Meanwhile, the high electronegativity (*χ*) of PDI-(CN)_2_ (5.17 eV) supports the powerful electron-withdrawing nature of cyano groups, making it the strongest electron acceptor in contrast to donors with lower electronegativity, such as PDI-(Py)_2_ (4.10 eV). This contrasting character is directly quantified by their IP/EA shifts relative to the parent PDI: the cyano groups deeply stabilize the system (ΔEA = 0.42 eV & ΔIP = 0.47 eV) as well as planar heteroatom-fused benzimidazole ring selectively stabilize the system's IP (ΔIP = 0.40 eV & ΔEA = −0.09. eV), whereas the pyrrolidine groups trigger a massive system destabilization (ΔIP = −0.92 eV & ΔEA = −0.37 eV), quantitatively confirming the synergistic impact of synergistic mesomeric donation and core twisting.

Within this perspective, interconnected effects starting with steric hindrance and core twist provide the basis for the S_o_ → S_1_ excitation and the corresponding dipole moment magnitude change to gain a partial CT character. Quantitative theoretical electronegativity, hardness and FMO analyses agree with HOMO destabilization and narrowing the HOMO–LUMO gap as is shown in agreement with the bathochromic shift that was observed experimentally. Hence, validating this mechanism gives us a rational design principle for tuning optical properties of extended π-conjugated scaffolds, serving as predictive models for larger carbon-based molecular architectures for light-harvesting applications.^11,46^

## 4 Conclusion

In this work, an interconnected investigation of some uniquely bay-functionalized PDIs was carried out by combining computational methods with targeted experimental spectroscopy. The results indicate that structural modification of the sterically hindered bay position is an effective approach to tailor the structural, electronic, and photophysical properties of the PDI core. The long-range-corrected (CAM-B3LYP-D3) computational model was selected and proved consistent with experimental spectroscopy, hence supporting the underlying structure–property relationships, specifically:

• Steric hindrance is predicted to induce a twist in the PDI core, reaching 21° for PDI-(Py)_2_. This geometric disruption synergizes with the strong mesomeric electron donation of the substituents leading to frontier molecular orbital modulation with a destabilized HOMO level (−6.02 eV), confirmed by the unique electrochemical oxidation.

• Partial CT character is further shown in the electron-rich PDI-(Py)_2_ the S_0_ → S_1_ transition as supported by a calculated (Δ*µ*) of 1.67 Debye (increasing by ∼46% to 2.44 D upon relaxation).

• The computational model successfully accounts for the contrasting optical behavior across the series. It predicts a significant bathochromic shift of >150 nm for twisted, electron-rich derivative PDI-(Py)_2_. Conversely, PDI-(CN)_2_ is governed by electron-withdrawing effects that deeply stabilize the HOMO level (−7.42 eV) being effectively planar with little to no spectral shift, while the planar, heteroatom-fused PDI-Imd shows selective HOMO stabilization and a blue-shift, consistent with experimental observations.

• Time-resolved spectroscopy further highlights distinct kinetic profiles across the series, with the bay twisted donor-subsituted PDI-(Py)_2_ showing the more complex excited-state relaxation, which is consistent with enhanced excited-state relaxation and contrasts theoretically with the profile of relatively rigid, more planar derivatives.

• This experimental and spectroscopic behavior is further substantiated by quantitative trends in DFT-derived descriptors, including chemical hardness, electronegativity, and ionization potentials.

In summary, pyrrolidinyl bay substitution proves most effective for red/NIR-shifted light-harvesting by successfully coupling steric core-twisting with nitrogen-donor electronics. In contrast, cyano substitution primarily enhances electron-accepting character, while benzimidazole fusion shifts the principal absorption toward higher energies. Ultimately, this foundational understanding uncovers the mechanistic basis of bay-functionalized PDI photophysics, serving as a practical springboard for the rational design and screening of perylene-based molecular nanographenes in next-generation optoelectronics.

## Conflicts of interest

The authors declare that they have no known competing financial interests.

## Supplementary Material

RA-016-D6RA02167K-s001

## Data Availability

The datasets examined and collected will be made available upon request and all supporting data for this study are also available in the Supplementary information (SI). Supplementary information is available. See DOI: https://doi.org/10.1039/d6ra02167k.
