# Supplementary material for: Modulating the photophysical properties of perylene-based light-harvesting materials via bay-induced distortion and core twisting: a combined computational and experimental study
Source: RSC Adv. 2026 Jul 2;16(34):32250–62. doi: 10.1039/d6ra02167k (PMC13326189; doi:10.1039/d6ra02167k)
Supplement: RA-016-D6RA02167K-s001 [file RA-016-D6RA02167K-s001.pdf]

Supplementary Information (SI) for RSC Advances.
This journal is © The Royal Society of Chemistry 2026

Supporting Information

Modulating the Photophysical Properties of Perylene-Based Light-Harvesting Materials via Bay-Induced Distortion and Core Twisting: A Combined Computational and Experimental Study

Yousef H. Arafa[a, b], Morad M. El-Hendawy[c,d]*, Ahmed Abdelmoneim[a], Mohamed E. El-Khouly[a]*

[a] *Nanoscience Program, Institute of Basic and Applied Science, Egypt-Japan University of Science and Technology, New Borg El-Arab city, Alexandria, Egypt*

[b] *Department of Chemistry, Faculty of Science, Sohag University, Sohag, 82524, Egypt*

[c] *School of Chemistry, University of the Witwatersrand, 2050 Johannesburg, South Africa*

[d] *Department of Chemistry, Faculty of Science, New Valley University, Kharga, Egypt*

Supporting Information

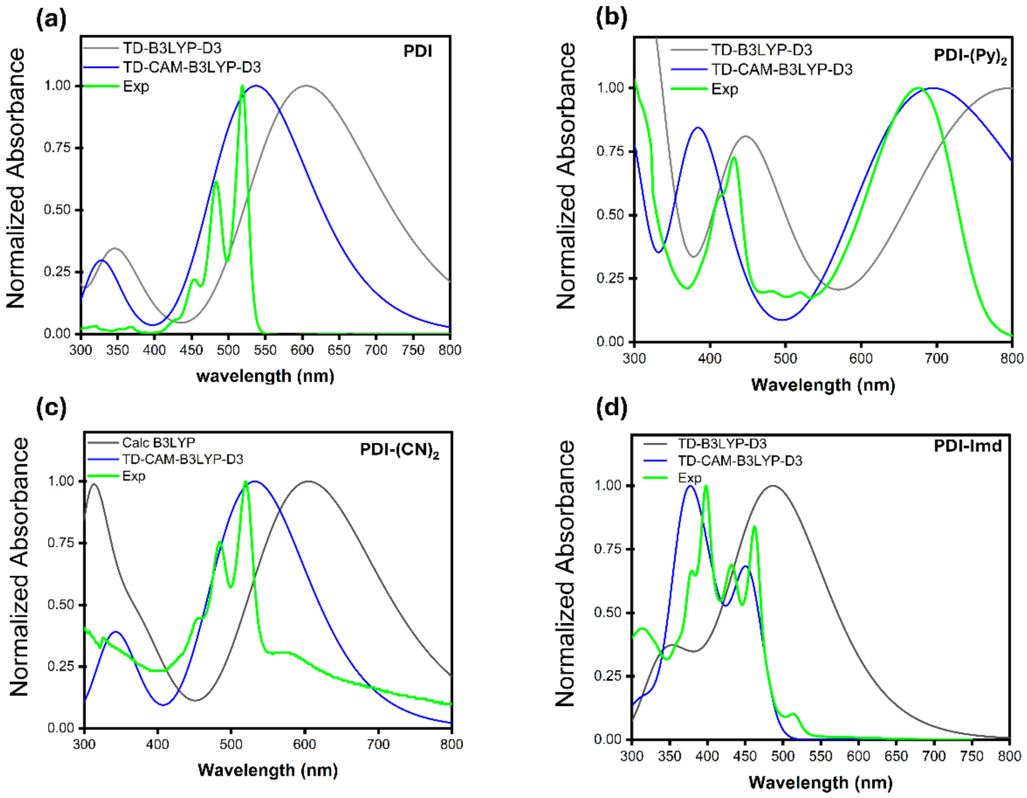

Figure S1. Overlay of the normalized experimental absorption spectra of the investigated structures **(a-d)** in Acetonitrile and in comparison, with TD-CAM-B3LYP-D3 and TD-B3LYP-D3 computed spectra.

Table S1. Cartesian coordinates (Å) of the of the investigated compounds optimized geometries at B3LYP-D3 level of theory and the 6-31g+(d) basis set employing acetonitrile solvent using SMD solvation model

Parent PDI

charge=0, multiplicity=1

C 0.83045300 1.18975400 -0.02221300
C -0.63527600 1.30433400 -0.02310100
C -1.42684300 0.11174000 -0.02307200
C -0.83044400 -1.18977100 -0.02208900
C 0.63528500 -1.30435100 -0.02301000
C 1.42685300 -0.11175700 -0.02311200
C -2.85171900 0.22306200 -0.02356400
C -3.65908600 -0.93881200 -0.02178800
C -3.06043100 -2.18913300 -0.01940700
C -1.66598000 -2.31030400 -0.02000600
C -1.28677600 2.54109500 -0.02359200
C -2.68305500 2.63797900 -0.02321100
C -3.46961300 1.49618300 -0.02356300
C 1.28678600 -2.54111200 -0.02339700

34 C 2.68306500 -2.63799700 -0.02305200
35 C 3.46962200 -1.49620000 -0.02354500
36 C 2.85172900 -0.22307900 -0.02364300
37 C 3.65909600 0.93879500 -0.02199600
38 C 3.06044100 2.18911500 -0.01968000
39 C 1.66599000 2.31028700 -0.02023600
40 C 4.94507600 -1.63004700 -0.02256300
41 N 5.70074700 -0.44372500 -0.03830700
42 C 5.13682500 0.83871700 -0.02258100
43 O 5.48885200 -2.72801800 -0.00988900
44 O 5.84999700 1.83580100 -0.01206500
45 C -4.94506600 1.63002800 -0.02255800
46 N -5.70073500 0.44370700 -0.03824400
47 C -5.13681400 -0.83873400 -0.02229000
48 O -5.84998300 -1.83582100 -0.01180600
49 O -5.48884900 2.72799800 -0.01004700
50 C 7.18000800 -0.56871800 -0.04074300
51 C -7.17999700 0.56871500 -0.04079000
52 C -7.89962200 -0.16730100 -1.21445800
53 C -8.92464800 -1.10481900 -0.54461900
54 C -9.22886500 -0.42247300 0.80163800
55 C -7.84925600 0.07295300 1.26623300
56 C 7.89967500 0.16719400 -1.21444900
57 C 8.92461000 1.10483600 -0.54464500
58 C 9.22879600 0.42262700 0.80168700
59 C 7.84918200 -0.07280000 1.26626400
60 H -3.68575900 -3.07714100 -0.01763400
61 H -1.24627700 -3.31012800 -0.01861300
62 H -0.71718400 3.46399100 -0.02406200
63 H -3.16194800 3.61265500 -0.02287800
64 H 0.71719400 -3.46400900 -0.02374800
65 H 3.16195700 -3.61267300 -0.02263400
66 H 3.68576700 3.07712400 -0.01800100
67 H 1.24628600 3.31011100 -0.01889400
68 H 7.35198500 -1.63858700 -0.13902000
69 H -7.35194300 1.63857800 -0.13919600
70 H -8.41143100 0.58108600 -1.83291000
71 H -7.19977900 -0.70207100 -1.86263300
72 H -8.46053400 -2.08124500 -0.36100800
73 H -9.81996800 -1.26257800 -1.15885300
74 H -9.90412400 0.43208900 0.64547000
75 H -9.70563100 -1.09138900 1.52917700
76 H -7.27994700 -0.76084900 1.68923100
77 H -7.89838700 0.86764000 2.02042300
78 H 8.41155600 -0.58123600 -1.83279000
79 H 7.19984700 0.70186400 -1.86272400
80 H 8.46042700 2.08125300 -0.36114500
81 H 9.81995100 1.26259700 -1.15884800
82 H 9.90409100 -0.43192500 0.64562600
83 H 9.70550700 1.09162900 1.52918400

H 7.27982900 0.76102800 1.68915100
H 7.89830200 -0.86741000 2.02053600

PDI-$(Py)_2$

charge=0, multiplicity=1

C 0.75033500 -1.21343400 0.01605500
C -0.68430000 -1.26780500 0.27981300
C -1.41425500 -0.05833700 0.05207900
C -0.75033300 1.21343000 0.01607000
C 0.68430400 1.26779900 0.27981100
C 1.41425700 0.05833200 0.05206600
C -2.81950500 -0.10601600 -0.17203400
C -3.53755800 1.05802200 -0.52362700
C -2.86053500 2.26683600 -0.64760300
C -1.49385100 2.34228800 -0.36928300
C -1.41038300 -2.45489200 0.60841500
C -2.80325900 -2.48325900 0.31697700
C -3.48584000 -1.35189100 -0.08379000
C 1.41038700 2.45489000 0.60840100
C 2.80326000 2.48325900 0.31694400
C 3.48583800 1.35189100 -0.08382800
C 2.81950600 0.10601400 -0.17205900
C 3.53755500 -1.05802100 -0.52366700
C 2.86052900 -2.26683100 -0.64766100
C 1.49384700 -2.34228500 -0.36932800
C 4.93649900 1.45236900 -0.36786300
N 5.61206200 0.25981300 -0.68514100
C 4.98633700 -0.99492100 -0.79051000
O 5.53093600 2.52466400 -0.32169000
O 5.63767400 -1.98855000 -1.10030600
C -4.93650800 -1.45236300 -0.36779200
N -5.61206600 -0.25981000 -0.68508800
C -4.98634000 0.99492200 -0.79046800
O -5.63770700 1.98857400 -1.10012800
O -5.53094900 -2.52465500 -0.32159500
C 7.06820300 0.34835700 -0.95543000
C -7.06821700 -0.34833400 -0.95532500
C -7.95928800 0.58327800 -0.07475000
C -8.79414400 1.41483700 -1.06987700
C -8.86802200 0.52822800 -2.32660800
C -7.45011900 -0.05773900 -2.42888300
C 7.95938200 -0.58295100 -0.07464000
C 8.79426400 -1.41471200 -1.06957500
C 8.86802100 -0.52843600 -2.32654900
C 7.45006900 0.05739700 -2.42892400
N 0.82231500 3.54554800 1.20323500
N -0.82231400 -3.54554800 1.20326000

C -0.29143300 3.42747800 2.16637300
C -0.05979600 4.57980300 3.15163500
C 0.60943500 5.64280500 2.26923000
C 1.56721000 4.80012300 1.42470300
C 0.29140900 -3.42746800 2.16642600
C 0.05975400 -4.57978900 3.15168700
C -0.60945300 -5.64279800 2.26927100
C -1.56721500 -4.80012000 1.42472500
H -3.40784000 3.15397100 -0.95308500
H -0.98804700 3.29621600 -0.47814700
H -3.36727900 -3.40213300 0.42137900
H 3.36727700 3.40213700 0.42132600
H 3.40783100 -3.15396200 -0.95316000
H 0.98804200 -3.29621100 -0.47820300
H 7.31407200 1.38391900 -0.72932700
H -7.31414800 -1.38382100 -0.72894800
H -8.61354300 -0.04299700 0.54535500
H -7.36797500 1.20673400 0.60150500
H -8.25868400 2.34132700 -1.30965100
H -9.78175000 1.68406700 -0.67480100
H -9.60018900 -0.27895400 -2.17430600
H -9.16589100 1.07801800 -3.22840900
H -6.77120200 0.68812300 -2.85403100
H -7.39193200 -0.96215500 -3.04668300
H 8.61361700 0.04354600 0.54526300
H 7.36815300 -1.20626800 0.60181400
H 8.25886600 -2.34130900 -1.30907600
H 9.78190700 -1.68375700 -0.67446700
H 9.60013300 0.27884300 -2.17449600
H 9.16589400 -1.07844700 -3.22821400
H 6.77118900 -0.68863500 -2.85383800
H 7.39178400 0.96163900 -3.04696800
H -0.28105500 2.43445700 2.63244500
H -1.26123000 3.54197200 1.66335000
H -0.99377500 4.91710900 3.61370900
H 0.62747700 4.26630000 3.94867300
H 1.13666200 6.41659200 2.83698900
H -0.13620300 6.13038900 1.62725100
H 1.83704500 5.26135300 0.46729300
H 2.49482900 4.60703700 1.98581900
H 0.28101200 -2.43444500 2.63249500
H 1.26122000 -3.54195700 1.66342900
H 0.99372400 -4.91709000 3.61378300
H -0.62753800 -4.26628600 3.94870900
H -1.13668800 -6.41658400 2.83702200
H 0.13620100 -6.13038000 1.62730800
H -1.83703600 -5.26135500 0.46731300
H -2.49484200 -4.60703000 1.98582600

PDI-$(CN)_2$

charge=0, multiplicity=1

C 1.18077900 0.81750100 0.09073600
C 1.30147700 -0.63066500 -0.11276600
C 0.11675600 -1.42149900 -0.00271600
C -1.18077900 -0.81750100 0.09073600
C -1.30147700 0.63066500 -0.11276600
C -0.11675600 1.42149900 -0.00271600
C 0.21463300 -2.84528500 0.02378500
C -0.92999500 -3.64023700 0.25934400
C -2.15257600 -3.02848400 0.47366900
C -2.27422400 -1.63513600 0.38401500
C 2.51183800 -1.30347100 -0.39614300
C 2.58115600 -2.71455500 -0.42914400
C 1.46082400 -3.47907100 -0.18353300
C -2.51183800 1.30347100 -0.39614300
C -2.58115600 2.71455500 -0.42914400
C -1.46082400 3.47907100 -0.18353300
C -0.21463300 2.84528500 0.02378500
C 0.92999500 3.64023700 0.25934400
C 2.15257600 3.02848400 0.47366900
C 2.27422400 1.63513600 0.38401500
C -1.58507000 4.95956700 -0.17486000
N -0.42166700 5.69957600 0.08670900
C 0.83524700 5.12112600 0.30188500
O -2.65883900 5.50649800 -0.38525700
O 1.81858200 5.81687200 0.51730600
C 1.58507000 -4.95956700 -0.17486000
N 0.42166700 -5.69957600 0.08670900
C -0.83524700 -5.12112600 0.30188500
O -1.81858200 -5.81687200 0.51730600
O 2.65883900 -5.50649800 -0.38525700
C -0.53667700 7.18090100 0.11002200
C 0.53667700 -7.18090100 0.11002200
C 0.04603400 -7.85535800 1.42940400
C -1.00950400 -8.89127500 0.99201700
C -0.61093700 -9.24167600 -0.45332500
C -0.21463300 -7.88106100 -1.05024900
C -0.04603400 7.85535800 1.42940400
C 1.00950400 8.89127500 0.99201700
C 0.61093700 9.24167600 -0.45332500
C 0.21463300 7.88106100 -1.05024900
C -3.73506500 0.64126100 -0.73775400
N -4.75472500 0.18174300 -1.05658600
C 3.73506500 -0.64126100 -0.73775400
N 4.75472500 -0.18174300 -1.05658600
H -3.02393300 -3.63682800 0.69614500

H -3.25082100 -1.20576300 0.56438300
H 3.52788600 -3.20166300 -0.64004000
H -3.52788600 3.20166300 -0.64004000
H 3.02393300 3.63682800 0.69614500
H 3.25082100 1.20576300 0.56438300
H -1.60349800 7.36242200 -0.00227300
H 1.60349800 -7.36242200 -0.00227300
H 0.90083500 -8.35448600 1.90296700
H -0.34646100 -7.13033600 2.14787600
H -2.00206000 -8.42484600 0.99331400
H -1.04317600 -9.76621000 1.65299500
H 0.25580000 -9.91932200 -0.45030200
H -1.41315000 -9.73345000 -1.01761200
H -1.11473900 -7.31677700 -1.31432100
H 0.41203000 -7.95891000 -1.94669800
H -0.90083500 8.35448600 1.90296700
H 0.34646100 7.13033600 2.14787600
H 2.00206000 8.42484600 0.99331400
H 1.04317600 9.76621000 1.65299500
H -0.25580000 9.91932200 -0.45030200
H 1.41315000 9.73345000 -1.01761200
H 1.11473900 7.31677700 -1.31432100
H -0.41203000 7.95891000 -1.94669800

PDI-Imd

charge=0, multiplicity=1

C 0.53287600 0.10977400 -2.48230800
C -0.92817300 0.04147400 -2.44075800
C -1.56944900 -0.29392000 -1.21030500
C -0.81185000 -0.56700900 -0.02904700
C 0.61441900 -0.50459400 -0.06955400
C 1.27244600 -0.16671300 -1.29296400
C -2.99111800 -0.36219000 -1.14980100
C -3.64605300 -0.69671700 0.07574300
C -2.91669800 -0.95906700 1.20655900
C -1.49406000 -0.90142800 1.17884800
C -1.73424100 0.29283400 -3.56477900
C -3.12272900 0.22343100 -3.49396200
C -3.75915200 -0.10100000 -2.29495300
C 1.38956900 -0.77678000 1.09947500
C 2.81263700 -0.70944100 1.03755400
C 3.44527900 -0.38244300 -0.13360100
C 2.69483700 -0.10497900 -1.31702300
C 3.36854000 0.22742400 -2.50253900
C 2.63648900 0.49681300 -3.65983400
C 1.24558800 0.43896100 -3.64849500

C 4.93319300 -0.31898900 -0.15203800
N 5.55010500 0.04523400 -1.35270800
C 4.84897900 0.30841300 -2.54381100
O 5.59129600 -0.56894000 0.84912900
O 5.43903600 0.60046100 -3.57589100
C -5.24075800 -0.16587400 -2.25244400
N -5.84557400 -0.49243600 -1.03099600
C -5.13046900 -0.76519500 0.14556700
O -5.70270700 -1.04665200 1.19044300
O -5.91472700 0.06132100 -3.24899300
C 7.04044200 0.18424900 -1.38854100
C -7.34028200 -0.53664200 -0.95180700
C -7.97203800 0.83666900 -1.24092000
C -7.89829600 -1.69647500 -1.79608200
C 7.53996300 1.27188800 -0.42044400
C 7.72113700 -1.18677200 -1.22739400
C 6.77831500 2.59841400 -0.53246500
C 7.37103300 3.70047600 0.35341600
C 6.59987300 5.02343500 0.27144600
C 7.18314000 6.12585200 1.16355500
C 6.41162000 7.44902600 1.08342700
C 7.00055400 8.54158800 1.98170400
C 9.22017700 -1.15949900 -1.55449000
C 9.85520200 -2.55559000 -1.52632800
C 11.35735500 -2.54288200 -1.83458200
C 11.99743300 -3.93617100 -1.80996000
C 13.49997800 -3.92318400 -2.11768300
C 14.12955600 -5.31972600 -2.09060600
C -9.37545100 -1.99783100 -1.50951300
C -9.87808900 -3.23654100 -2.26200600
C -11.35737900 -3.54542800 -2.00251200
C -11.86308600 -4.78292500 -2.75437400
C -13.34180000 -5.08289900 -2.48836600
C -7.33408500 1.98797100 -0.45351700
C -8.06482800 3.32107700 -0.65176000
C -7.41542500 4.48572600 0.10552300
C -8.14424200 5.82098500 -0.08751200
C -7.49238600 6.98742100 0.66545400
C -8.22913300 8.31592200 0.46618700
C -0.70304800 -1.16793800 2.31741000
C 0.68867700 -1.10702700 2.28068500
C -1.08288300 -1.54043700 3.67968500
N 0.08963500 -1.69096800 4.41233600
C 1.23927600 -1.44039300 3.63799900
O 2.38138000 -1.49689200 4.03627100
N -2.16787600 -1.76235000 4.36660300
C -1.70101700 -2.08597700 5.65366300
C -0.27937500 -2.04664800 5.70508200
C -2.42951100 -2.41691100 6.79870000
C -1.71480500 -2.70001300 7.96598100

321 C -0.30833600 -2.65533400 7.99588900
322 C 0.44121000 -2.32642600 6.86045500
323 H -3.42483000 -1.21358200 2.13148300
324 H -1.28363600 0.54666200 -4.51806200
325 H -3.72676700 0.42203900 -4.37422800
326 H 3.39763600 -0.91834800 1.92604700
327 H 3.16657700 0.75505000 -4.57154300
328 H 0.71847400 0.65969300 -4.57051300
329 H 7.23784200 0.53373800 -2.40351700
330 H -7.53114500 -0.77027300 0.09713200
331 H -7.93469300 1.05121200 -2.31456000
332 H -9.03271600 0.76169100 -0.96570500
333 H -7.76237400 -1.48044400 -2.86148300
334 H -7.30086600 -2.59285800 -1.57246700
335 H 7.50381100 0.90573200 0.61128100
336 H 8.59708600 1.44784800 -0.66080400
337 H 7.56821000 -1.55981900 -0.20854000
338 H 7.21943900 -1.89276600 -1.90584600
339 H 6.77368800 2.93188000 -1.58211100
340 H 5.72457200 2.44644000 -0.25603900
341 H 7.38865800 3.35380800 1.39896500
342 H 8.42175700 3.87220200 0.07090800
343 H 6.58405700 5.37094500 -0.77394200
344 H 5.54847400 4.84837100 0.55035700
345 H 7.19860500 5.77800200 2.20898800
346 H 8.23480500 6.30182900 0.88575000
347 H 6.39877400 7.79828200 0.03937000
348 H 5.36054500 7.27259200 1.35972000
349 H 6.43060200 9.47755100 1.90575500
350 H 6.99552300 8.23222000 3.03649900
351 H 8.04195900 8.76152200 1.70704200
352 H 9.37149200 -0.70976600 -2.54865300
353 H 9.74905500 -0.51277800 -0.83980100
354 H 9.68987400 -3.00991700 -0.53653300
355 H 9.33935100 -3.20531300 -2.25096700
356 H 11.52142400 -2.08464500 -2.82297600
357 H 11.87175000 -1.89354200 -1.10828200
358 H 11.83365400 -4.39523400 -0.82174400
359 H 11.48403600 -4.58623700 -2.53659400
360 H 13.66349300 -3.46496900 -3.10535600
361 H 14.01280100 -3.27433200 -1.39090100
362 H 15.20468100 -5.28231000 -2.31276800
363 H 14.00856900 -5.78954200 -1.10421100
364 H 13.65828100 -5.98105400 -2.83147900
365 H -9.51818300 -2.14480600 -0.42718200
366 H -9.99672000 -1.13459200 -1.78822500
367 H -9.71998000 -3.09318000 -3.34274100
368 H -9.26797200 -4.10828300 -1.97701200
369 H -11.51590500 -3.68770000 -0.92144500
370 H -11.96737200 -2.67330400 -2.28772400

H -11.70514400 -4.63983600 -3.83453900
H -11.25374800 -5.65438700 -2.46902600
H -13.67837800 -5.97222400 -3.03831500
H -13.52333100 -5.26283700 -1.41926000
H -13.97808500 -4.24032900 -2.79422900
H -7.31582100 1.73434800 0.61798700
H -6.28336900 2.10689600 -0.75675600
H -8.09875800 3.55960900 -1.72677300
H -9.11273000 3.21503500 -0.32924700
H -7.37657800 4.24423300 1.17977000
H -6.36856400 4.59210000 -0.22104300
H -8.18598900 6.06125700 -1.16209400
H -9.19049200 5.71611400 0.24190300
H -7.45059400 6.74738200 1.73915300
H -6.44752500 7.09305100 0.33480500
H -7.74195300 9.13385700 1.01415900
H -8.25876500 8.59680200 -0.59615600
H -9.26795700 8.24951000 0.81941600
H -3.51582700 -2.45060700 6.77235200
H -2.25655700 -2.96064300 8.87230700
H 0.21199900 -2.88213400 8.92365800
H 1.52622900 -2.29054700 6.87671000

Table S2. Cartesian coordinates (Å) of the of the investigated compounds optimized geometries as radical anions and ferrocene/ferrocenium at the B3LYP-D3 level of theory and the 6-311++g(d,p) basis set employing acetonitrile solvent using SMD solvation model

Parent PDI$^{\cdot -}$

charge=-1, multiplicity=2

C 0.81777900 1.18722400 -0.02093600
C -0.62559900 1.29884000 -0.02359100
C -1.41959700 0.10992900 -0.02253800
C -0.81778600 -1.18720200 -0.02072400
C 0.62559100 -1.29881900 -0.02341900
C 1.41959000 -0.10990700 -0.02257900
C -2.85186900 0.22052600 -0.02294800
C -3.65292100 -0.94434400 -0.01896700
C -3.03861400 -2.20297300 -0.01455100
C -1.66324800 -2.32213600 -0.01601700
C -1.28712400 2.55013600 -0.02677900
C -2.66435300 2.64386900 -0.02752700
C -3.46596700 1.49471400 -0.02572300
C 1.28711500 -2.55011500 -0.02644300
C 2.66434400 -2.64384800 -0.02724000
C 3.46595900 -1.49469300 -0.02565200
C 2.85186100 -0.22050400 -0.02302900
C 3.65291400 0.94436600 -0.01923900
C 3.03860700 2.20299600 -0.01498600
C 1.66324100 2.32215900 -0.01642800
C 4.92091200 -1.62852400 -0.02534700
N 5.67437900 -0.43610900 -0.03086900
C 5.11098500 0.85085100 -0.01830900
O 5.48374500 -2.72662900 -0.02111000
O 5.84284900 1.84339200 -0.00955700
C -4.92092200 1.62854300 -0.02532500
N -5.67438700 0.43612400 -0.03077200
C -5.11099400 -0.85083000 -0.01803500
O -5.84286800 -1.84336200 -0.00913700
O -5.48375600 2.72664900 -0.02142200
C 7.15079700 -0.56124100 -0.03217400
C -7.15080200 0.56123600 -0.03213700
C -7.87432000 -0.15650300 -1.21222200
C -8.91742400 -1.08146500 -0.55660700
C -9.20944400 -0.41589400 0.79982700
C -7.82592000 0.06032600 1.26781900
C 7.87437300 0.15652600 -1.21220500
C 8.91749100 1.08142000 -0.55651400
C 9.20945300 0.41577200 0.79989600
C 7.82590500 -0.06043700 1.26783100
H -3.66454000 -3.08691000 -0.01043600
H -1.23663100 -3.31554900 -0.01291900

H -0.71350700 3.46669800 -0.02896400
H -3.14576500 3.61408700 -0.02967400
H 0.71349800 -3.46667700 -0.02846800
H 3.14575600 -3.61406600 -0.02927300
H 3.66453500 3.08693200 -0.01101300
H 1.23662400 3.31557200 -0.01345900
H 7.32198400 -1.62883900 -0.11903300
H -7.32201400 1.62883300 -0.11895000
H -8.36759800 0.59852500 -1.83191400
H -7.17892500 -0.70137900 -1.84979100
H -8.47466600 -2.06650300 -0.38940500
H -9.81381600 -1.20916700 -1.16985000
H -9.87662800 0.44344800 0.65918200
H -9.68794300 -1.09029500 1.51533600
H -7.26717400 -0.77944700 1.68285800
H -7.87123000 0.84877800 2.02354600
H 8.36763300 -0.59848700 -1.83193000
H 7.17900800 0.70146200 -1.84975700
H 8.47477000 2.06646900 -0.38928000
H 9.81390700 1.20911000 -1.16972400
H 9.87662000 -0.44357900 0.65922800
H 9.68794900 1.09012600 1.51545100
H 7.26717900 0.77931900 1.68292500
H 7.87118800 -0.84894600 2.02350100

PDI-$(Py)_2^{\bullet -}$

charge=-1, multiplicity=2

C 0.74706200 -1.21199900 0.01738600
C -0.67667000 -1.26660400 0.26255300
C -1.41003900 -0.05698800 0.05154600
C -0.74704800 1.21198900 0.01739400
C 0.67668200 1.26659100 0.26257200
C 1.41005200 0.05697800 0.05155900
C -2.82326200 -0.11132900 -0.17270000
C -3.54198300 1.05769400 -0.51248700
C -2.85521800 2.27515800 -0.62260600
C -1.50438500 2.35291700 -0.35154600
C -1.40879800 -2.46459600 0.58266900
C -2.78313900 -2.49246600 0.33185400
C -3.48405100 -1.35554700 -0.08639100
C 1.40880400 2.46458000 0.58271000
C 2.78315000 2.49245100 0.33192500
C 3.48406800 1.35553800 -0.08632700
C 2.82327700 0.11132300 -0.17267400
C 3.54200200 -1.05769400 -0.51247500
C 2.85523800 -2.27515800 -0.62261300
C 1.50440200 -2.35292000 -0.35156700
C 4.91573800 1.45691000 -0.35255100

N 5.59339500 0.26117500 -0.67383000
C 4.97348200 -0.99855400 -0.77437900
O 5.52783200 2.52978200 -0.29629900
O 5.64519500 -1.99077200 -1.07690300
C -4.91571500 -1.45691800 -0.35264800
N -5.59337500 -0.26117400 -0.67388900
C -4.97346300 0.99856100 -0.77439500
O -5.64515000 1.99076200 -1.07702900
O -5.52780600 -2.52979400 -0.29644300
C 7.04760200 0.35391500 -0.93771300
C -7.04757100 -0.35392000 -0.93782800
C -7.94301100 0.55480800 -0.04121700
C -8.80427700 1.37929300 -1.01714000
C -8.86759700 0.51225800 -2.28709900
C -7.44355200 -0.05184800 -2.40347400
C 7.94295500 -0.55508500 -0.04129200
C 8.80422000 -1.37937000 -1.01738600
C 8.86763600 -0.51202400 -2.28712600
C 7.44362500 0.05218500 -2.40341900
N 0.77261300 3.57006100 1.14256200
N -0.77262700 -3.57008100 1.14253400
C -0.21634600 3.40144700 2.23452800
C -0.04739600 4.65390500 3.10639500
C 0.51887400 5.68853100 2.12483600
C 1.48917100 4.84099500 1.30559800
C 0.21628500 -3.40147600 2.23454500
C 0.04729600 -4.65393800 3.10639700
C -0.51893300 -5.68855700 2.12480800
C -1.48919300 -4.84101400 1.30553400
H -3.40561600 3.15950300 -0.92033900
H -0.99939900 3.30523900 -0.43805700
H -3.35292400 -3.39729000 0.48300600
H 3.35293300 3.39727200 0.48310000
H 3.40563900 -3.15949700 -0.92035700
H 0.99941700 -3.30524100 -0.43809900
H 7.28571000 1.39064100 -0.72506400
H -7.28563400 -1.39070900 -0.72543500
H -8.57598000 -0.08274600 0.58380800
H -7.35242600 1.18464100 0.62316100
H -8.29451500 2.31863600 -1.24522100
H -9.79274600 1.61582500 -0.61326000
H -9.58682400 -0.30428600 -2.14738300
H -9.17423000 1.07232900 -3.17489200
H -6.77961500 0.70447000 -2.82370900
H -7.38141600 -0.94781100 -3.02680700
H 8.57592800 0.08227700 0.58392500
H 7.35230300 -1.18505400 0.62289700
H 8.29441900 -2.31863100 -1.24572100
H 9.79266000 -1.61605400 -0.61352400
H 9.58690000 0.30444800 -2.14717800

H 9.17427600 -1.07189100 -3.17504700
H 6.77966700 -0.70399100 -2.82387400
H 7.38156400 0.94830800 -3.02652900
H -0.02805700 2.47299800 2.78220200
H -1.23128200 3.34378000 1.83167000
H -0.98725400 4.96070500 3.56961000
H 0.67941500 4.46713900 3.90322700
H 1.01490000 6.52746500 2.61685800
H -0.27522600 6.08393100 1.48331900
H 1.73677700 5.26893700 0.33054000
H 2.42737100 4.71022200 1.86655200
H 0.02797400 -2.47303000 2.78221700
H 1.23124000 -3.34380800 1.83173300
H 0.98713400 -4.96074300 3.56965200
H -0.67955000 -4.46717600 3.90319900
H -1.01498100 -6.52749300 2.61680300
H 0.27519400 -6.08395400 1.48332200
H -1.73676000 -5.26895000 0.33046400
H -2.42741700 -4.71024200 1.86644900

PDI-$(CN)_2^{-\cdot}$

Charge=-1, multiplicity=2

C 1.23723000 0.71450400 0.02813400
C 1.24648800 -0.71604800 -0.16621900
C 0.00882300 -1.41737300 -0.05199900
C -1.23723000 -0.71450400 0.02813400
C -1.24648800 0.71604800 -0.16621900
C -0.00882300 1.41737300 -0.05199900
C -0.00030800 -2.85001800 0.00046900
C -1.20025400 -3.54737100 0.23358000
C -2.38743600 -2.83171600 0.42445000
C -2.40546300 -1.45529900 0.31896100
C 2.41882600 -1.48994500 -0.44942600
C 2.37962100 -2.88842100 -0.44396300
C 1.20499200 -3.57358300 -0.18033500
C -2.41882600 1.48994500 -0.44942600
C -2.37962100 2.88842100 -0.44396300
C -1.20499200 3.57358300 -0.18033500
C 0.00030800 2.85001800 0.00046900
C 1.20025400 3.54737100 0.23358000
C 2.38743600 2.83171600 0.42445000
C 2.40546300 1.45529900 0.31896100
C -1.22422800 5.04133300 -0.13972200
N -0.00161600 5.68671000 0.11895200
C 1.21651900 5.01311800 0.30656800
O -2.25552300 5.68618100 -0.31932000
O 2.24525000 5.64843700 0.52429400
C 1.22422800 -5.04133300 -0.13972200

N 0.00161600 -5.68671000 0.11895200
C -1.21651900 -5.01311800 0.30656800
O -2.24525000 -5.64843700 0.52429400
O 2.25552300 -5.68618100 -0.31932000
C -0.00882300 7.16911400 0.17829100
C 0.00882300 -7.16911400 0.17829100
C -0.53249200 -7.77866100 1.50723100
C -1.64356200 -8.76010800 1.08790100
C -1.26574700 -9.16318000 -0.34828000
C -0.77672500 -7.84647100 -0.97048400
C 0.53249200 7.77866100 1.50723100
C 1.64356200 8.76010800 1.08790100
C 1.26574700 9.16318000 -0.34828000
C 0.77672500 7.84647100 -0.97048400
C -3.66088200 0.92612000 -0.85793700
N -4.68860300 0.54029800 -1.22925800
C 3.66088200 -0.92612000 -0.85793700
N 4.68860300 -0.54029800 -1.22925800
H -3.29618400 -3.37358500 0.65405800
H -3.34007200 -0.94518900 0.49320600
H 3.27982200 -3.45088700 -0.65617800
H -3.27982200 3.45088700 -0.65617800
H 3.29618400 3.37358500 0.65405800
H 3.34007200 0.94518900 0.49320600
H -1.05712000 7.42914900 0.07947600
H 1.05712000 -7.42914900 0.07947600
H 0.28306600 -8.31255500 2.00388400
H -0.89070400 -7.01404400 2.19583300
H -2.60421800 -8.23927200 1.07768300
H -1.72659900 -9.61638900 1.76270800
H -0.45070800 -9.89693600 -0.32924200
H -2.09660600 -9.60785000 -0.90258700
H -1.63234200 -7.23339700 -1.25658000
H -0.14845300 -7.98969700 -1.85321900
H -0.28306600 8.31255500 2.00388400
H 0.89070400 7.01404400 2.19583300
H 2.60421800 8.23927200 1.07768300
H 1.72659900 9.61638900 1.76270800
H 0.45070800 9.89693600 -0.32924200
H 2.09660600 9.60785000 -0.90258700
H 1.63234200 7.23339700 -1.25658000
H 0.14845300 7.98969700 -1.85321900

PDI-Imd $^{\cdot-}$

charge=-1, multiplicity=2

C 0.52724300 0.16732700 -2.46521800
C -0.91391900 0.09725000 -2.42872000
C -1.56224300 -0.28381700 -1.21694500

C -0.80691900 -0.59948200 -0.04768300
C 0.61084600 -0.53292500 -0.08173300
C 1.27078200 -0.14614300 -1.28933300
C -2.99049400 -0.35115200 -1.16494500
C -3.64464900 -0.73469900 0.04316100
C -2.90668600 -1.04439100 1.17087500
C -1.50267900 -0.98491100 1.15300500
C -1.72449400 0.39410800 -3.55662300
C -3.09722200 0.32457400 -3.49444400
C -3.75098100 -0.04455000 -2.30408600
C 1.39671400 -0.84741700 1.08441000
C 2.79493700 -0.77141600 1.02609200
C 3.44296500 -0.39161400 -0.14249500
C 2.69957000 -0.07507800 -1.31091700
C 3.36918500 0.30396700 -2.49055600
C 2.62049900 0.60575200 -3.64151400
C 1.24524400 0.54122700 -3.63238900
C 4.91014100 -0.32134200 -0.15507000
N 5.52483900 0.10502400 -1.34136200
C 4.82778100 0.39504300 -2.53158000
O 5.58849300 -0.61199400 0.83013800
O 5.43581900 0.71701100 -3.55530700
C -5.21478900 -0.10051300 -2.27091900
N -5.82284600 -0.45102100 -1.05446900
C -5.11234100 -0.80230600 0.10731300
O -5.70329500 -1.14521000 1.13066900
O -5.90183200 0.15361000 -3.26001900
C 7.01048100 0.26040100 -1.36973800
C -7.31498200 -0.46216300 -0.96748200
C -7.92627700 0.91997000 -1.25155900
C -7.90371100 -1.60863800 -1.80560900
C 7.51344700 1.28122200 -0.33504200
C 7.70376600 -1.11029400 -1.30518300
C 6.73570300 2.60061100 -0.33496200
C 7.32201000 3.63629000 0.62889200
C 6.53281400 4.94862800 0.66251000
C 7.11534000 5.98790700 1.62501000
C 6.32437600 7.29953100 1.66224900
C 6.91497000 8.32915300 2.62869200
C 9.19673300 -1.05542200 -1.64722000
C 9.83840900 -2.44528600 -1.71347800
C 11.33452000 -2.40908700 -2.04002800
C 11.97848400 -3.79715300 -2.10890900
C 13.47488400 -3.76290700 -2.43604100
C 14.10648700 -5.15566100 -2.50165400
C -9.37948100 -1.88932400 -1.50123000
C -9.91148900 -3.11802300 -2.24629300
C -11.38969300 -3.40556100 -1.96717100
C -11.92496000 -4.63465300 -2.70888600
C -13.40266600 -4.91183000 -2.42175300

C -7.25994400 2.06231300 -0.47889300
C -7.95188700 3.41133200 -0.69498400
C -7.27492000 4.56549600 0.05066600
C -7.96469400 5.91697500 -0.15796500
C -7.28630300 7.07198800 0.58582400
C -7.98399400 8.41710200 0.36947700
C -0.70768600 -1.28997400 2.28121500
C 0.67840300 -1.22691600 2.25547300
C -1.09847900 -1.70936900 3.62838600
N 0.06569800 -1.88333400 4.36301700
C 1.22024400 -1.60555100 3.59051900
O 2.35829700 -1.68223400 3.99684900
N -2.18375500 -1.95611200 4.30412000
C -1.72713600 -2.32156300 5.58074400
C -0.30745000 -2.28158500 5.63913100
C -2.45998600 -2.69000700 6.70690500
C -1.75507800 -3.00962000 7.86769300
C -0.35248800 -2.96346700 7.90738900
C 0.40170100 -2.59722900 6.78982900
H -3.42147400 -1.33474500 2.07705500
H -1.26558000 0.68327800 -4.49192600
H -3.69794000 0.55583200 -4.36526500
H 3.38411500 -1.00838000 1.90020800
H 3.14840700 0.89450100 -4.54192600
H 0.71284400 0.78583100 -4.54121100
H 7.20150100 0.67782000 -2.35662000
H -7.50463800 -0.69164600 0.07930800
H -7.89829600 1.12972500 -2.32254500
H -8.98031300 0.86504700 -0.95977300
H -7.77635400 -1.39011200 -2.86820300
H -7.32023500 -2.51213800 -1.59189400
H 7.49744200 0.84206600 0.66412600
H 8.56009000 1.48975500 -0.58071100
H 7.56328000 -1.54534100 -0.31308400
H 7.20075400 -1.77259000 -2.01989800
H 6.71767600 3.01652900 -1.35064200
H 5.69105400 2.41336700 -0.06185100
H 7.35520000 3.20863400 1.63949500
H 8.36296800 3.84625300 0.34938900
H 6.49448400 5.37360900 -0.34922000
H 5.49351400 4.73647300 0.94532100
H 7.15452300 5.56236200 2.63656800
H 8.15477100 6.20203200 1.34224600
H 6.28565000 7.72559400 0.65190300
H 5.28619200 7.08549600 1.94492700
H 6.33126000 9.25512700 2.63570200
H 6.93593700 7.94123200 3.65293900
H 7.94323300 8.58585400 2.35127100
H 9.33304200 -0.54731000 -2.61112800
H 9.72987800 -0.45448900 -0.90197500

H 9.68935700 -2.95761800 -0.75410200
H 9.31746900 -3.04892000 -2.46800300
H 11.48274100 -1.89393700 -2.99832200
H 11.85496400 -1.80646800 -1.28398400
H 11.83092800 -4.31279900 -1.15070800
H 11.45841600 -4.40072000 -2.86460200
H 13.62237600 -3.24862200 -3.39394700
H 13.99490700 -3.16042500 -1.68077700
H 15.17430000 -5.10238200 -2.73649200
H 14.00153500 -5.68125500 -1.54628300
H 13.62656800 -5.77002200 -3.27122100
H -9.50870500 -2.03679000 -0.42089000
H -9.98973400 -1.01993800 -1.77061200
H -9.76773700 -2.97595000 -3.32537200
H -9.31320800 -3.99588500 -1.96980700
H -11.53395700 -3.54529100 -0.88756200
H -11.98853500 -2.52808900 -2.24537600
H -11.78142700 -4.49488000 -3.78759600
H -11.32721500 -5.51157400 -2.43074500
H -13.75892700 -5.79373200 -2.96351700
H -13.57029000 -5.08722100 -1.35350500
H -14.02797400 -4.06305700 -2.71950200
H -7.25191700 1.82467100 0.59260200
H -6.21005700 2.14911600 -0.78118400
H -7.97306200 3.63747500 -1.76917700
H -8.99983000 3.33843600 -0.37529900
H -7.24717800 4.33528900 1.12385600
H -6.22874200 4.63953900 -0.27373000
H -7.99311000 6.14724200 -1.23135900
H -9.01106400 5.84421200 0.16758100
H -7.25808500 6.84237800 1.65829900
H -6.24122100 7.14511800 0.26019800
H -7.47916600 9.22349300 0.91080700
H -7.99856600 8.68707400 -0.69210900
H -9.02263900 8.38271300 0.71615700
H -3.54267500 -2.72404600 6.67227200
H -2.30040500 -3.29950200 8.75868800
H 0.16088500 -3.21819600 8.82773200
H 1.48285000 -2.56122300 6.81996000

Ferrocene

charge=0, multiplicity=1

C -1.66582000 1.19934600 -0.14947400
C -1.66503100 0.51280100 1.09471900
C -1.66559600 0.22816500 -1.18689200
C -1.66403800 -0.88261400 0.82628700
C -1.66433400 -1.05856900 -0.58378700
C 1.66554000 0.22685300 -1.18714600

C 1.66410900 -1.05925000 -0.58277000
C 1.66598200 1.19909000 -0.15075100
C 1.66398300 -0.88185500 0.82710900
C 1.66521200 0.51383000 1.09415300
H -1.63923800 -1.67043600 1.56390000
H -1.64085600 0.97090800 2.07189200
H -1.63992300 -2.00356200 -1.10510900
H -1.64214400 0.43217100 -2.24667000
H -1.64263100 2.27034500 -0.28297300
H 1.63957300 -2.00476800 -1.10311300
H 1.64204700 0.42983300 -2.24713100
H 1.64292300 2.26994300 -0.28539800
H 1.64121300 0.97295800 2.07085000
H 1.63913800 -1.66890100 1.56554800
Fe -0.00000600 0.00056600 -0.00040300

Ferrocenium

charge=1, multiplicity=2

C -1.76428000 1.19049200 -0.14231300
C -1.80669200 0.49374200 1.09310400
C -1.69594100 0.22711200 -1.19076600
C -1.76551700 -0.89765300 0.82039900
C -1.69909300 -1.07006400 -0.59170900
C 1.68809300 0.43447400 -1.13727100
C 1.72960900 -0.93991000 -0.75415200
C 1.72571400 1.21966400 0.05475300
C 1.79505000 -0.99652200 0.66501500
C 1.79241400 0.33083400 1.16177600
H -1.73769500 -1.68585300 1.55794600
H -1.79977700 0.94731500 2.07316800
H -1.63902500 -2.01294300 -1.11423200
H -1.64083900 0.44022500 -2.24765100
H -1.74245300 2.26290300 -0.26357700
H 1.68547200 -1.78804000 -1.42090700
H 1.62694300 0.81255500 -2.14638100
H 1.68366900 2.29737900 0.10818400
H 1.77981400 0.61528800 2.20341600
H 1.78466100 -1.89538500 1.26305100
Fe 0.00011800 0.00205900 0.00438300

Table S3. Cartesian coordinates (Å) of the investigated compounds relaxed first singlet excited state (S_1) geometries optimized at the CAM-B3LYP-D3 level of theory and the 6-311++G(d,p) basis set employing acetonitrile solvent using the SMD solvation model

Parent PDI

charge=0, multiplicity=1

C 0.80918300 1.17832300 -0.06193500
C -0.61550000 1.29025900 -0.04850300
C -1.40153400 0.11021800 -0.05163000
C -0.80919800 -1.17830300 -0.06182000
C 0.61548600 -1.29023800 -0.04836800
C 1.40151900 -0.11019600 -0.05161000
C -2.81982800 0.22096100 -0.04054000
C -3.61729000 -0.93169800 -0.05206200
C -3.02323800 -2.19445100 -0.07661900
C -1.65761500 -2.31794700 -0.08059100
C -1.27660700 2.54807000 -0.02584900
C -2.64459900 2.63830600 -0.00589400
C -3.42916000 1.48365500 -0.01365700
C 1.27659300 -2.54804600 -0.02557700
C 2.64458500 -2.63827800 -0.00560900
C 3.42914700 -1.48362800 -0.01348600
C 2.81981300 -0.22093700 -0.04050100
C 3.61727500 0.93172000 -0.05212800
C 3.02322300 2.19447100 -0.07680900
C 1.65760000 2.31796600 -0.08080700
C 4.89473400 -1.61424500 0.01502900
N 5.64830300 -0.44081600 -0.01825400
C 5.08524800 0.83078300 -0.03657300
O 5.43027000 -2.71066100 0.06293000
O 5.79356500 1.82466600 -0.04105400
C -4.89474800 1.61427800 0.01484100
N -5.64831600 0.44084300 -0.01831200
C -5.08526400 -0.83075600 -0.03652100
O -5.79359400 -1.82463000 -0.04097700
O -5.43027600 2.71070100 0.06269600
C 7.12209700 -0.56510100 -0.00806200
C -7.12210800 0.56509400 -0.00811000
C -7.84335900 -0.14145100 -1.18554100
C -8.86776900 -1.07882500 -0.53289100
C -9.15716900 -0.42531200 0.82226200
C -7.77798400 0.04764000 1.28511900
C 7.84337400 0.14132600 -1.18554900
C 8.86785400 1.07867600 -0.53297300
C 9.15722500 0.42524000 0.82222400
C 7.77802000 -0.04760800 1.28512800
H -3.65476600 -3.07231500 -0.09029600
H -1.23071800 -3.30945200 -0.10035800

H -0.70148400 3.46220600 -0.01783000
H -3.13054000 3.60407800 0.01645700
H 0.70147200 -3.46218100 -0.01746400
H 3.13052700 -3.60404800 0.01684500
H 3.65475100 3.07233300 -0.09056200
H 1.23070200 3.30946900 -0.10067100
H 7.29592600 -1.63197900 -0.08314800
H -7.29598200 1.63196000 -0.08326000
H -8.34977600 0.61567900 -1.78821800
H -7.14949900 -0.66881100 -1.83822700
H -8.41554800 -2.05980500 -0.37289200
H -9.76392900 -1.21149600 -1.14218600
H -9.82344000 0.43354500 0.68788000
H -9.63038600 -1.10362700 1.53499600
H -7.21345100 -0.79640800 1.68204800
H -7.81811800 0.82292600 2.05231900
H 8.34973900 -0.61587400 -1.78818100
H 7.14954000 0.66868500 -1.83826400
H 8.41570000 2.05969700 -0.37303900
H 9.76401700 1.21124500 -1.14228700
H 9.82344700 -0.43366300 0.68789600
H 9.63048800 1.10357700 1.53490800
H 7.21354000 0.79649500 1.68201600
H 7.81812100 -0.82285100 2.05237200

PDI-$(Py)_2$

charge=0, multiplicity=1

C 0.73686200 -1.20703700 -0.03246800
C -0.66509800 -1.24899200 0.22390200
C -1.39338100 -0.05410900 0.01177000
C -0.73683700 1.20701800 -0.03251300
C 0.66511200 1.24897600 0.22391300
C 1.39340500 0.05408900 0.01182800
C -2.79429900 -0.11422800 -0.21931900
C -3.50208600 1.03716100 -0.55352900
C -2.82584300 2.26056600 -0.68629600
C -1.48514500 2.34705200 -0.42860500
C -1.39660500 -2.44711700 0.56427900
C -2.76790500 -2.50065200 0.23638900
C -3.44651700 -1.36859400 -0.15510400
C 1.39660500 2.44710600 0.56430100
C 2.76792200 2.50063300 0.23647700
C 3.44654700 1.36857300 -0.15498300
C 2.79433000 0.11420600 -0.21921500
C 3.50212500 -1.03718000 -0.55341600
C 2.82588700 -2.26058700 -0.68619000
C 1.48518400 -2.34707400 -0.42852600
C 4.88787800 1.45941200 -0.42056500

N 5.56514000 0.26932600 -0.70192000
C 4.94151300 -0.97494500 -0.80883800
O 5.48133400 2.52975800 -0.38932500
O 5.59532800 -1.96716900 -1.10226200
C -4.88784800 -1.45942700 -0.42068700
N -5.56510900 -0.26933400 -0.70201400
C -4.94147700 0.97493400 -0.80893800
O -5.59531100 1.96718100 -1.10224300
O -5.48130800 -2.52977100 -0.38945200
C 7.02281500 0.34929500 -0.93125000
C -7.02280700 -0.34927100 -0.93120600
C -7.87816300 0.57762600 -0.02830200
C -8.73772900 1.40754500 -0.99089700
C -8.84781400 0.52885300 -2.24123500
C -7.44185400 -0.05687900 -2.38386500
C 7.87831200 -0.57747000 -0.02834300
C 8.73780500 -1.40745500 -0.99094700
C 8.84771400 -0.52888900 -2.24139000
C 7.44171500 0.05676800 -2.38392300
N 0.81744900 3.46937600 1.23068700
N -0.81747900 -3.46937600 1.23070700
C -0.32065500 3.32733200 2.16253600
C -0.05933500 4.39939000 3.21332100
C 0.59267000 5.50813200 2.39141000
C 1.53780400 4.72568300 1.49102900
C 0.32058600 -3.32731800 2.16260300
C 0.05922700 -4.39936800 3.21338800
C -0.59274400 -5.50811900 2.39146100
C -1.53784400 -4.72568000 1.49103500
H -3.38333900 3.13251200 -1.00243900
H -0.97416700 3.29177500 -0.55633800
H -3.32770100 -3.41480600 0.35552100
H 3.32771200 3.41479000 0.35561800
H 3.38339000 -3.13253200 -1.00232300
H 0.97421200 -3.29179800 -0.55626700
H 7.26827800 1.37958300 -0.70224500
H -7.26828800 -1.37952700 -0.70207300
H -8.51225100 -0.04356900 0.60856000
H -7.26541300 1.19750000 0.62405000
H -8.21386600 2.33186800 -1.24289800
H -9.70952500 1.67015900 -0.56785600
H -9.57358200 -0.27364600 -2.07049500
H -9.16678100 1.08034500 -3.12805800
H -6.77597100 0.68545600 -2.82419500
H -7.40666300 -0.95702300 -3.00052800
H 8.51244700 0.04382200 0.60837600
H 7.26566700 -1.19730000 0.62414700
H 8.21395200 -2.33182600 -1.24279300
H 9.70965900 -1.66998500 -0.56798700
H 9.57346800 0.27365900 -2.07081700

H 9.16660500 -1.08046100 -3.12819100
H 6.77581200 -0.68564300 -2.82409800
H 7.40641400 0.95684400 -3.00068000
H -0.35277400 2.31268600 2.55951500
H -1.26449400 3.52511500 1.65441600
H -0.97881600 4.70711300 3.71098000
H 0.63939400 4.03033700 3.96867900
H 1.12502100 6.24329600 2.99412500
H -0.15976400 6.02839600 1.79322000
H 1.77590700 5.22784600 0.55271700
H 2.47382700 4.51399300 2.01789400
H 0.35268400 -2.31266800 2.55957400
H 1.26444600 -3.52510200 1.65452500
H 0.97868900 -4.70708400 3.71108400
H -0.63953300 -4.03031000 3.96871500
H -1.12511600 -6.24327900 2.99416100
H 0.15971500 -6.02838700 1.79330500
H -1.77591000 -5.22785400 0.55272000
H -2.47388700 -4.51398400 2.01786100

PDI-$(CN)_2$

charge=0, multiplicity=1

C 1.22436400 -0.71556500 0.14730100
C 0.00113000 -1.42761900 -0.03772000
C -1.21118300 -0.70195000 0.05136400
C -1.22436400 0.71556500 0.14730100
C -0.00113000 1.42761900 -0.03772000
C 1.21118300 0.70195000 0.05136400
C -2.45256300 -1.39610700 0.06047700
C -3.65372200 -0.70542200 0.26858100
C -3.63507500 0.66488200 0.51553500
C -2.45256300 1.35811300 0.45379100
C -0.08623700 -2.83614600 -0.30761100
C -1.30599500 -3.48380900 -0.34029600
C -2.48462000 -2.78331300 -0.12620200
C 0.08623700 2.83614600 -0.30761100
C 1.30599500 3.48380900 -0.34029600
C 2.48462000 2.78331300 -0.12620200
C 2.45256300 1.39610700 0.06047700
C 3.65372200 0.70542200 0.26858100
C 3.63507500 -0.66488200 0.51553500
C 2.45256300 -1.35811300 0.45379100
C 3.76135900 3.52349600 -0.15046600
N 4.92818200 2.79625700 0.06714500
C 4.94405800 1.42179100 0.27577400
O 3.78378600 4.72525400 -0.34988000
O 5.99300800 0.83008200 0.45462600
C -3.76135900 -3.52349600 -0.15046600

N -4.92818200 -2.79625700 0.06714500
C -4.94405800 -1.42179100 0.27577400
O -5.99300800 -0.83008200 0.45462600
O -3.78378600 -4.72525400 -0.34988000
C 6.21383200 3.52971600 0.05252400
C -6.21383200 -3.52971600 0.05252400
C -7.06900700 -3.36838000 1.33614300
C -8.43841200 -2.87277600 0.85293800
C -8.53220700 -3.38839000 -0.58663200
C -7.12212100 -3.15937800 -1.13371900
C 7.06900700 3.36838000 1.33614300
C 8.43841200 2.87277600 0.85293800
C 8.53220700 3.38839000 -0.58663200
C 7.12212100 3.15937800 -1.13371900
C -1.03762900 3.63189900 -0.67668800
N -1.89811400 4.31652100 -1.01514200
C 1.03762900 -3.63189900 -0.67668800
N 1.89811400 -4.31652100 -1.01514200
H -4.55881800 1.17567600 0.75019200
H -2.46688100 2.41275700 0.67835500
H -1.35321500 -4.54323000 -0.55209100
H 1.35321500 4.54323000 -0.55209100
H 4.55881800 -1.17567600 0.75019200
H 2.46688100 -2.41275700 0.67835500
H 5.92220500 4.56887300 -0.04343700
H -5.92220500 -4.56887300 -0.04343700
H -7.16456800 -4.34518500 1.81505200
H -6.60982600 -2.69440700 2.05763400
H -8.45232900 -1.78079400 0.84873600
H -9.25753400 -3.22137500 1.48483100
H -8.76511900 -4.45855700 -0.58545500
H -9.29921300 -2.88247700 -1.17618200
H -6.99460400 -2.10834100 -1.39427000
H -6.89354200 -3.75979500 -2.01591900
H 7.16456800 4.34518500 1.81505200
H 6.60982600 2.69440700 2.05763400
H 8.45232900 1.78079400 0.84873600
H 9.25753400 3.22137500 1.48483100
H 8.76511900 4.45855700 -0.58545500
H 9.29921300 2.88247700 -1.17618200
H 6.99460400 2.10834100 -1.39427000
H 6.89354200 3.75979500 -2.01591900

PDI-Imd

charge=0, multiplicity=1

C 0.55224500 -0.37885600 -2.46276500
C -0.90423000 -0.44504100 -2.41502200
C -1.54751800 -0.55710500 -1.15482100

C -0.79901100 -0.62768400 0.05214200
C 0.62904600 -0.56413400 0.00639900
C 1.28542800 -0.43370800 -1.24852100
C -2.96496400 -0.59752300 -1.09408500
C -3.62123200 -0.72283400 0.15722500
C -2.90034600 -0.80279000 1.31208500
C -1.48306000 -0.75537600 1.28694900
C -1.69809100 -0.39479100 -3.56368300
C -3.08119200 -0.42130900 -3.48990600
C -3.72126300 -0.51345100 -2.26521200
C 1.39915200 -0.62763300 1.19427300
C 2.81084600 -0.54770900 1.12269400
C 3.44305800 -0.41021800 -0.07985700
C 2.70064300 -0.35282700 -1.28326700
C 3.36979500 -0.21993100 -2.50554500
C 2.64336700 -0.17559600 -3.68179800
C 1.25894000 -0.25298100 -3.65998200
C 4.92088700 -0.32495700 -0.10797900
N 5.53611700 -0.14662000 -1.34465400
C 4.84193700 -0.11363600 -2.55580900
O 5.57510900 -0.38348600 0.91235300
O 5.43145400 -0.01135100 -3.61226800
C -5.19767900 -0.52826100 -2.21623200
N -5.80668700 -0.65879100 -0.96924300
C -5.09909000 -0.77300800 0.22722900
O -5.67336700 -0.90433200 1.28768300
O -5.86265700 -0.41559800 -3.22409000
C 7.01760000 0.02459100 -1.40131200
C -7.29698400 -0.66307000 -0.88601700
C -7.89469800 0.65563200 -1.38647200
C -7.87241600 -1.91942000 -1.54655700
C 7.49108700 1.21841400 -0.56501200
C 7.72028900 -1.29659700 -1.07646800
C 6.69336000 2.49725700 -0.80836500
C 7.25719000 3.69623400 -0.05062700
C 6.44448700 4.97206600 -0.25617700
C 6.99570400 6.17178700 0.50996100
C 6.18037200 7.44708500 0.30834800
C 6.73627900 8.63722700 1.08487900
C 9.20949100 -1.28204800 -1.41640800
C 9.86252200 -2.64985800 -1.23071800
C 11.35397600 -2.65392700 -1.55647000
C 12.00747000 -4.02228800 -1.38079600
C 13.49934700 -4.02907600 -1.70658000
C 14.14005900 -5.40262400 -1.52955000
C -9.34952800 -2.14244200 -1.22621100
C -9.87260000 -3.46205100 -1.78933900
C -11.35240800 -3.69694400 -1.49795800
C -11.87947400 -5.01415000 -2.06267600
C -13.35952500 -5.23774500 -1.76616200

1106 C -7.24713200 1.89394700 -0.77089500
1107 C -7.96282800 3.18636500 -1.15398900
1108 C -7.31386200 4.43185600 -0.55559600
1109 C -8.03605900 5.72618600 -0.92072600
1110 C -7.38816900 6.97241800 -0.32155500
1111 C -8.12082600 8.25914800 -0.69040600
1112 C -0.70319500 -0.83083100 2.45530500
1113 C 0.70309600 -0.76864300 2.41680700
1114 C -1.08181600 -0.97594800 3.83092100
1115 N 0.07913000 -1.00140500 4.57127100
1116 C 1.24528300 -0.87595300 3.77105000
1117 O 2.39047200 -0.86732000 4.20378000
1118 N -2.18260200 -1.09328800 4.57935500
1119 C -1.71010000 -1.19926800 5.86224400
1120 C -0.29329600 -1.14576000 5.89426600
1121 C -2.42663500 -1.34703000 7.05853200
1122 C -1.70187100 -1.43814800 8.25812700
1123 C -0.32087200 -1.38466900 8.26724200
1124 C 0.42433000 -1.23494900 7.06601800
1125 H -3.41681000 -0.90176100 2.25758000
1126 H -1.23993900 -0.32493400 -4.54004800
1127 H -3.67674200 -0.36977000 -4.39222600
1128 H 3.39548600 -0.59642900 2.03031900
1129 H 3.16951500 -0.07377000 -4.62213600
1130 H 0.73022400 -0.20657800 -4.60175200
1131 H 7.21194700 0.25729900 -2.44627100
1132 H -7.49506900 -0.73135300 0.18150100
1133 H -7.83479700 0.70511100 -2.47499700
1134 H -8.95649800 0.64174600 -1.12330000
1135 H -7.73121000 -1.86980700 -2.62790900
1136 H -7.29744600 -2.78032400 -1.18663300
1137 H 7.47468700 0.96539100 0.49590500
1138 H 8.53624800 1.39537100 -0.83637500
1139 H 7.58215600 -1.53834100 -0.02032700
1140 H 7.23060800 -2.08848500 -1.65458900
1141 H 6.67609800 2.71863900 -1.88282700
1142 H 5.64999200 2.34784900 -0.50870800
1143 H 7.29563400 3.45992000 1.02025200
1144 H 8.29389800 3.87106900 -0.36427700
1145 H 6.40990600 5.21040400 -1.32680400
1146 H 5.40673900 4.79226600 0.05197900
1147 H 7.03173100 5.93249100 1.58046500
1148 H 8.03308200 6.35433500 0.20167100
1149 H 6.14860100 7.68879400 -0.76067300
1150 H 5.14320800 7.26282300 0.61260300
1151 H 6.13616500 9.53750200 0.92402600
1152 H 6.74954700 8.43318300 2.16043500
1153 H 7.76305000 8.86173200 0.77830500
1154 H 9.34490900 -0.95246700 -2.45423800
1155 H 9.72968300 -0.55090400 -0.78860000

H 9.71703100 -2.98217500 -0.19519700
H 9.35069200 -3.38482200 -1.86443300
H 11.49953300 -2.31310700 -2.58936900
H 11.86586500 -1.92361600 -0.91715500
H 11.86262800 -4.36342900 -0.34775000
H 11.49563600 -4.75336500 -2.01960500
H 13.64418000 -3.68695000 -2.73819200
H 14.01081200 -3.30036800 -1.06657000
H 15.20756100 -5.38157100 -1.76730100
H 14.03507000 -5.75545800 -0.49846200
H 13.66763200 -6.14376500 -2.18236400
H -9.49573100 -2.12733700 -0.13885600
H -9.94948900 -1.32091600 -1.63144400
H -9.70989700 -3.48129400 -2.87428600
H -9.28519400 -4.29128500 -1.37570500
H -11.51608300 -3.67822200 -0.41290100
H -11.93944100 -2.86683500 -1.91112800
H -11.71572700 -5.03221300 -3.14667700
H -11.29312800 -5.84342800 -1.64954700
H -13.71344800 -6.18607800 -2.18077600
H -13.54610100 -5.25506000 -0.68749000
H -13.97144400 -4.43744200 -2.19475000
H -7.23524300 1.79467500 0.32150600
H -6.19914300 1.95922200 -1.08504900
H -7.98427700 3.27812400 -2.24725600
H -9.00940300 3.13124500 -0.82938100
H -7.28027800 4.33194700 0.53671400
H -6.27059600 4.49230100 -0.89044500
H -8.06994700 5.82717500 -2.01306400
H -9.07948700 5.66603000 -0.58583500
H -7.35285100 6.87024400 0.76956600
H -6.34651200 7.03390300 -0.65827900
H -7.63782200 9.13651800 -0.25057700
H -8.14462400 8.40058700 -1.77579900
H -9.15680600 8.23659200 -0.33703600
H -3.50875700 -1.39001300 7.04947900
H -2.23294600 -1.55344900 9.19421100
H 0.20610500 -1.45892500 9.20990300
H 1.50534900 -1.19352500 7.0711800
